# Cancer patient-derived organoids: Novel models for the study of natural products

**DOI:** 10.7150/ijbs.114373

**Published:** 2025-07-11

**Authors:** Shuxin Liu, Ren Zhang, Yachen Liu, Xian Lin, Jian Chen

**Affiliations:** 1 Research Centre of Basic Integrative Medicine, School of Basic Medical Sciences, Guangzhou University of Chinese Medicine. Guangzhou 510006, China.; 2 Department of Thoracic Surgery, Ruijin Hospital, Shanghai Jiao Tong University School of Medicine, Shanghai 200025, China.; 3 Peking University Shenzhen Hospital; Shenzhen Peking University-The Hong Kong University of Science and Technology Medical Center; Shenzhen Key Laboratory of Inflammatory and Immunology Diseases; Shenzhen 518036, China.

**Keywords:** cancer patient-derived organoids, natural products, novel models, activity evaluation

## Abstract

Organoids are multicellular *in vitro* organ models that are self-organized and derived from stem cells or primary tissues in specific three-dimensional (3D) environments. Cancer patient-derived organoids (CPDOs) retain key characteristics of the original tumor, including genomic, epigenetic, and metabolic profiles, while accurately recapitulating the human tumor microenvironment (TME) - closely mirroring features observed in patient tumors. Compared to traditional cell lines and animal models, CPDOs offer significant advantages, making them increasingly valuable for cancer research and precision medicine. Meanwhile, natural products (NPs) remain a rich and pharmacologically promising source of anticancer drug candidates. In this review, we systematically summarized the important applications of different CPDOs in the efficacy evaluation, drug screening, and mechanism studies of NPs. Moreover, we also discussed the advantages, limitations, and future perspectives of CPDOs, proving valuable insights for researchers and clinicians in this field.

## 1. Introduction

Cancer is one of the diseases with the highest morbidity and mortality rates in the world. According to the latest estimates from the International Agency for Research on Cancer (IARC), approximately 20% of men or women in all regions of the world developed cancer in 2022, with men having a higher cancer mortality rate than women 1. In 2022, the top 5 cancers with the highest incidence rates in descending order are lung cancer, female breast cancer, colorectal cancer, prostate cancer, and gastric cancer 1. Lung cancer is known as the most common cancer and the leading cause of cancer morbidity and mortality all over the world. Nearly 2.5 million new cases and more than 1.8 million deaths of lung cancer were estimated to occur in 2022 1. In women, breast cancer is the most commonly diagnosed cancer and the leading cause of cancer mortality 1. Projections indicate that the burden of breast cancer will increase significantly by 2025, with persistent yet varied disparities and differences 2. Colorectal cancer represents one of the most prevalent and deadly cancers globally, accounting for approximately one in ten of all cancer cases and deaths in 2022 1. Among men, prostate cancer ranks as the second most common malignancy, with nearly 1.5 million new cases and nearly 397,000 deaths reported in 2022 1. Gastric cancer, with an estimated over 968,000 new cases and nearly 660,000 deaths in 2022, ranks fifth in both global incidence and mortality 1. Consequently, developing effective therapies for diverse types of cancer represents an urgent research priority.

Organoids are multicellular *in vitro* organ models that are self-organized and derived from stem cells or primary tissues in specific three-dimensional (3D) environments 3. Cancer patient-derived organoids (CPDOs) are able to maintain key features of the primary tumor, such as genomic, epigenetic, and metabolic properties, and CPDOs can mimic the human-specific tumor microenvironment (TME), which are very similar to those of tumors in cancer patients 4. Compared to patient-derived cancer cells (PDCs), CPDOs better maintain tumor heterogeneity and microenvironment features while being more cost-effective and time-efficient than patient-derived xenografts (PDXs) 5.

Early in 2011, Sato *et al.* 6 established patient-derived organoids by optimizing the previous human colon culture systems 7. Since then, CPDOs have been widely adopted in cancer research, leading to substantial progress in the field. Currently, diverse CPDOs have been successfully established using various methodological approaches 8, 9. These organoid systems have demonstrated considerable potential for clinical applications, including anticancer drug screening 10, 11, cancer chemoresistance research 12, 13, and modeling of cancer 14, 15.

Natural products (NPs) are rich and invaluable sources for anticancer drug discovery. Over the past few decades, more and more compounds from NPs such as paclitaxel 16-18, camptothecin 19-21, vincristine 22, 23, curcumin 24, 25, resveratrol 26, quercetin 27, aqueous extract of taxus chinensis var. mairei 28, grape seed procyanidin extract 29, *et al.* have been applied to treat different kinds of cancers. Chen's group also reported that a series of natural compounds including artemether 30, artemisitene 31-33, xanthotoxol 34, oxypalmatine 35, liensinine diperchlorate 32, *et al.* have good activity for inhibiting various cancer cells. NPs have advantages in huge scaffold diversity and chemical structural complexity compared with conventional synthetic molecules. In addition, chemically modified or structurally optimized NPs may have superior therapeutic effectiveness or absorption, distribution, metabolism, excretion and toxicity (ADMET) properties 36. Therefore, further development and utilization of NPs is very necessary and of great significance.

In the past, conventional animal and cell models have been used in drug screening and activity evaluation. However, animal and cell models also have many limitations, such as the long modeling time of animal models, high cost, large species differences between animal and human immune systems, and the inability of cell models to reflect the overall efficacy and side effects of drugs. As innovative and ideal preclinical models, CPDOs overcome the limitations of traditional animal and cell-based systems, serving as powerful tools for drug efficacy evaluation and high-throughput screening.

## 2. Construction Methods of CPDOs

**Figure 1:**
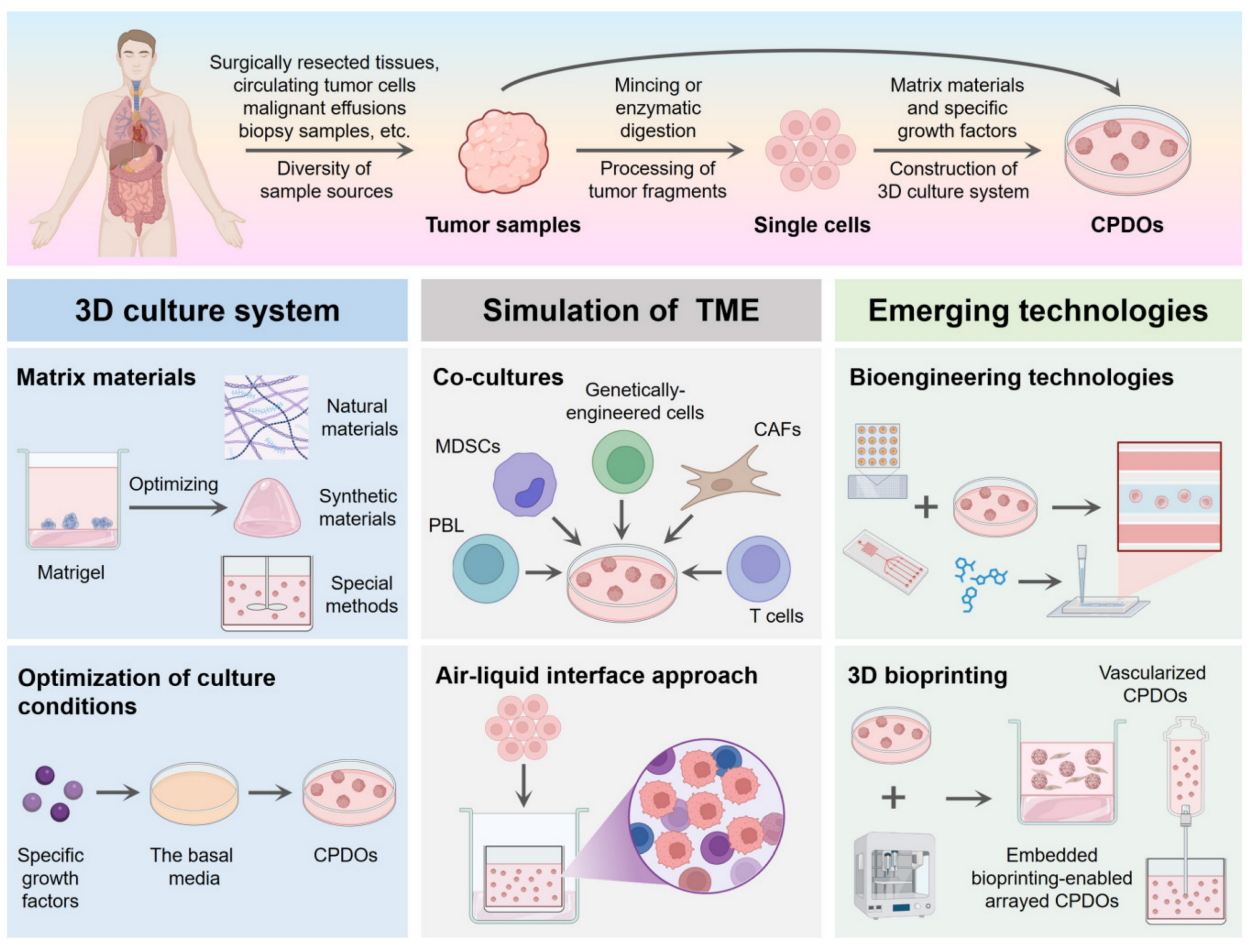
Construction methods of CPDOs. Tumor samples derived from patients can be processed to generate CPDOs. The 3D culture system of CPDOs generally relies on a combination of matrix materials and specific growth factors. Co-cultures and an air-liquid interface approach can better allow CPDOs to mimic the tumor microenvironment of the original tumor. CPDOs can also be combined with bioengineering technologies and 3D bioprinting to help overcome the limitations that exist in CPDOs. This figure was created with the help of BioRender (https://www.biorender.com/).

Currently, various types of CPDOs have been successfully established for biomedical research applications. This section systematically summarizes the established culture methodologies (Figure 1) and key biological characteristics of representative CPDOs, along with their commonly reported molecular markers (Table 1).

### 2.1 Sample acquisition and preprocessing

The establishment of CPDOs begins with the acquisition of patient tumor tissue samples, and common sources include surgically resected tissues, biopsy samples, and malignant effusions. Back in 2014, Gao *et al.* reported that CPDOs derived from patients with advanced prostate cancer could be successfully cultured from biopsy specimens and circulating tumor cells 82. In 2015, Sylvia *et al.* successfully generated pancreatic CPDOs from resected tumors and biopsies and exhibited ductal- and disease stage-specific features 83. Additionally, Gao *et al.* successfully established gastric CPDOs from endoscopic biopsies and surgical tissues of patients with gastric adenocarcinoma 84. By low-coverage whole-genome profiling, the study demonstrated that gastric CPDOs generated from endoscopic biopsies showed absence of tumor heterogeneity, and can thus be considered accurate models of human gastric cancer 84. Moreover, ovarian CPDOs can be successfully generated from patient-derived ovarian tumor tissues, ascites, and pleural fluid 85. A review in 2023 summarized the diversity of sample sources for lung CPDOs. Lung CPDOs are primarily derived from surgically resected tumor tissues, but can also be established from malignant pleural effusion or biopsy tissues 86.

In particular, high-quality tumor samples need to be processed rapidly to maintain cell viability and avoid changes in gene expression. As early as 2013, DeRose *et al.* reported that surgically removed tumor tissues from breast cancer patients could be processed in many different ways 87. They successfully cultured CPDOs by processing tumor fragments by mincing and enzymatic digestion with collagenase and hyaluronidase 87. Currently, a protocol has been developed for the rapid generation of patient-derived glioblastoma organoids from fresh tumor specimens 80. This method does not require the mechanical or enzymatic dissociation of the resected tumor tissues into single cells, and the addition of components such as serum to the culture medium 80.

### 2.2 Construction of 3D culture system

The culture of CPDOs generally relies on a combination of matrix materials and specific growth factors.

#### 2.2.1 Matrigel

At present, Matrigel is the most commonly used matrix for the culture of CPDOs. In 2016, Hubert *et al.* described a novel culture system that can generate patient-derived glioblastoma organoids 88. This system encapsulated the hypoxic gradients and cancer stem cell heterogeneity of tumors found *in vivo*. In 2020, Karakasheva *et al.* described protocols to generate and characterize esophageal CPDOs 89. Tumor tissue specimens were subjected to enzymatic and mechanical disruption to obtain single-cell suspensions, which were embedded in Matrigel and cultured in the unique organoid growth media 89. In 2020, Shi *et al.* successfully cultured lung CPDOs by resuspending tumor cells in 100% growth factor-reduced Matrigel and using Advanced DMEM/F12 basal media with additional supplements 40. In another study, Kim *et al.* developed a culture protocol in Matrigel using minimum basal media 42. Although this medium contained fewer reagents and growth factors compared to other protocols, lung CPDOs were still successfully established from tumor tissues or biopsy samples 42.

However, Matrigel is not well defined and is an animal-derived matrix, which is difficult to translate into clinical settings 90. Therefore, it is necessary to find new and alternative matrices, such as hydrogels generated from natural materials (e.g., fibronectin 91, collagen 92, hyaluronic acid 93) and synthetic materials 94, 95. In a study by Mosquera *et al.*, a synthetic polymer-based hydrogel platform was developed to culture prostate CPDOs that were initially derived in Matrigel and exposed to a week of defined extracellular matrix conditions in synthetic hydrogels 96. In 2022, Below *et al.* described a poly-ethylene glycol-based hydrogel system, incorporating the fibronectin-mimetic peptide *PHSRN-K-RGD*, the *GFOGER* peptide, and a basement membrane binding peptide to support cell adhesion 97. The hydrogels could mimic complex cell-extracellular matrix interactions, and the combination of three peptide anchors could also significantly enhance the number and size of pancreatic CPDOs 97. Recently, Cruz-Acuña *et al.* described a modular, tumor extracellular matrix-mimetic hydrogel platform that has defined physicochemical properties 98. This engineered hydrogel system not only supported robust *in vitro* growth and expansion of CPDOs derived from esophageal adenocarcinoma, but also had the potential to be adapted for the generation of different CPDOs 98. In addition, a mass-culture method for colorectal CPDOs was been devised that combined suspension culture and medium agitation using a rotating vessel 99. This protocol suspended and maintained the organoids in a culture medium supplemented with a certain concentration of Matrigel 99.

#### 2.2.2 Optimization of culture conditions

The composition of the culture media is critical for the growth and maintenance of CPDOs. In addition to the basal media, which contain the nutrients and essential ingredients for cell growth, the formulation of the media needs to be adjusted according to the type of tumor and the purpose of research. For example, specific growth factors, cytokines, and small molecules are added to the media to meet the growth needs of tumor cells in CPDOs and maintain their tumor characteristics.

A culture system was designed and developed that allowed long-term expansion of colorectal CPDOs in 2011 6. In the media that met the condition for stem cell culture, the concentration of Wnt3A, SB202190, nicotinamide, and dibenzazepine needed to be adjusted according to different differentiation conditions 6. In 2015, Wetering *et al.* utilized the Wnt-dependency of normal colonic stem cells to selectively expand colorectal CPDOs that could be successfully cultured in Human Intestinal Stem Cell medium minus Wnt 100. Based on the generic organoid medium (containing Advanced DMEM/F12 medium, epidermal growth factor, Noggin as well as the Wnt agonist R-spondin 1) developed by Sato *et al.* 101, Drost *et al.* successfully established prostate CPDOs and supported the long-term growth by continuing to append different compounds and growth factors 55. These additives could adjust and optimize the culture conditions of CPDOs, including B27 supplement, nicotinamide, N-acetylcysteine, A83-01, dihydrotestosterone, fibroblast growth factor 2 (FGF2), FGF10, prostaglandin E2, SB202190, Y-27632 55. In 2017, Broutier *et al.* optimized human liver expansion protocols, in order to selectively expand three of the most common subtypes of primary liver CPDOs 61. They designed a newly defined isolation medium, which consisted of the classical isolation medium without R-spondin-1, Noggin, and Wnt3a but supplied with Dexamethasone and Rho-kinase inhibitor 61. In 2018, Nuciforo *et al.* optimized the culture conditions of liver CPDOs, which allowed to generate long-term organoid cultures from needle biopsies of different patients with primary liver cancer 65. For example, they removed forskolin, N-acetyl-L-cysteine, nicotinamide, and hepatocyte growth factor (HGF) and added FGF19 to promote the growth of liver CPDOs 65. Besides, the mitogen Neuregulin 1, inhibition of Rho-associated coiled-coil containing protein kinase (ROCK), and the specific ROCK inhibitor Y-27632, were key additions in the culture media for the generation and long-term proliferation of breast CPDOs 46. In 2020, Maenhoudt *et al.* defined a culture medium that could strongly enhance the formation efficiency of epithelial ovarian CPDOs 75. After testing, neuregulin-1 was identified as the key component in the culture media for the development and growth of ovarian CPDOs 75. In 2021, Dekkers *et al.* developed a highly versatile protocol for the long-term culture of breast CPDOs 48. Adding specific medium components to the basal media including Wnt3a, hydrocortisone, β-estradiol, and forskolin, could enhance the growth characteristics of some CPDOs 48. Recently, Senkowski *et al.* proposed a protocol for long-term culture of high-grade serous ovarian CPDOs from cryopreserved tissues, achieving a markedly higher success rate than previously reported 77. The addition of epidermal growth factor (EGF), heregulin β-1, hydrocortisone, and forskolin to the basal media could further improve the growth and expansion of these CPDOs 77.

Therefore, it is necessary to continuously optimize the culture conditions of CPDOs, so that different types of CPDOs can be cultured successfully. However, the optimal culture conditions for the media and the specific concentration of supplements in the media need to be explored and determined in experiments.

### 2.3 Simulation of the tumor microenvironment

The physiological structure of CPDOs is not identical to that of intact human organs, and CPDOs lack direct connections with other human tissues 102. Hence, CPDOs can appropriately recapitulate the tumor immune microenvironment of the original tumor by preserving endogenous stromal components, or by appending exogenous immune cells, cancer-associated fibroblasts (CAFs) as well as other components 103. In 2018, Tsai *et al.* constructed complex 3D in-vitro models that included pancreatic CPDOs, CAFs, and T lymphocytes 69. The CPDOs were co-cultured with stromal and immune components of the tumors, facilitating the study of tumor-stroma and tumor-immune interaction 69. Additionally, co-cultures of peripheral blood lymphocytes (PBL) with CPDOs could induce patient-specific tumor-reactive T cell responses 104. Dijkstra *et al.* demonstrated that the co-culture of CPDOs with PBL enriched for tumor-reactive T cells from peripheral blood of patients with mismatch repair deficient colorectal and non-small cell lung cancer 104. Furthermore, co-cultures of CPDOs and chimeric antigen receptor T (CAR T) cells could better investigate patient-specific responses to immunotherapies 81. In 2020, Jacob *et al.* developed an optimized protocol to co-culture CAR T cells with patient-derived glioblastoma organoids, in order to mimic CAR T cell invasion into solid tumors 81. Recently, co-culture models of liver CPDOs with CAFs had been established, which could mimic the *in vivo* tumor settings and better study the cell-cell interactions in the tumor microenvironment 105. Liu *et al.* successfully established a 3D co-culture system of liver CPDOs with CAFs and demonstrated that CAFs could promote the growth of CPDOs in co-cultures 105. Moreover, Zhao *et al.* revealed that the co-culture of CPDOs derived from oral squamous cell carcinoma with CAFs increased the size and forming efficiency of organoids 106. It has been demonstrated that the co-culture of CPDOs with cytotoxic T lymphocytes (CTLs) and myeloid-derived suppressor cells (MDSCs), can allow for a more comprehensive investigation of the tumor microenvironment and the response of cancer to immunotherapy 107. In 2021, Koh *et al.* reported a CPDO/immune cell co-culture system that could be used to study PD-L1/PD-1 blockade and the immunosuppressive function of MDSCs, in order to develop immunotherapy for gastric cancer 107.

In addition to co-culture systems, an air-liquid interface (ALI) approach could be developed to more accurately reflect the tumor microenvironment in patients. In 2018, Neal *et al.* successfully established and expanded diverse CPDOs by inoculating mechanically dissociated tissue fragments in Type I collagen matrix ALI culture, and utilizing WENR basal media supplemented with specific growth factors 108.

These culture platforms of CPDOs can be used to mimic the immunotherapy responses of cancer patients and have great significance preclinical testing of immunotherapy.

### 2.4 Emerging technologies

Bioengineering technologies, including microwell array chips and microfluidic technology, can perform CPDOs-based drug screening and testing in a short time to predict specific drug responses of patients in clinical settings.

In 2021, Hu *et al.* employed an integrated superhydrophobic microwell array chip to derive large numbers of CPDOs from patient samples and enabled the CPDOs to maintain the 3D structures of parental tumor tissues 41. This microwell array-based approach could measure the responses of lung CPDOs to chemotherapeutic drugs in the nanoliter scale and shorten the drug sensitivity test to one week 41. In 2022, Ding *et al.* utilized droplet emulsion microfluidics with temperature control and dead-volume minimization to rapidly generate substantial Micro-Organospheres from tumor patient tissue samples 109. The core principle of droplet-based microfluidics was that suspended tumor cells were added to Matrigel, followed by mixing with a biphasic liquid to generate Micro-Organospheres 109. Recently, Kheiri *et al.* developed a multi-layer microfluidic platform that allowed high-fidelity formation and selective release of breast CPDOs with different shapes, for the study of cancer cell invasion in unconstrained environments 110. This microfluidic platform employed sliding microwells with different shapes as templates to generate CPDOs and utilized complementary microfluidic ducts to create a continuous fluidic path through the device 110. In addition, Choi *et al.* developed and characterized novel microfluidic culture systems for pancreatic CPDOs and demonstrated that these microfluidic devices had considerable advantages for personalized treatment based on cancer biopsies 111. The microfluidic device incorporated a port for direct injection of organoids or organoid fragments, which improved the efficiency of organoid/tissue utilization 111.

The utilization of novel technologies such as 3D bioprinting systems and artificial intelligence can optimize the workflow for the construction of CPDOs. CPDOs with emerging technologies enable the creation of more complex and practical models that can overcome the limitations of existing disease models. In 2023, Choi *et al.* utilized 3D bioprinting technology to develop vascularized lung CPDOs, which contained stromal cells, lung fibroblasts, and perfusable vessels 112. These advanced *in vitro* lung CPDOs recapitulated pulmonary fibrosis and more accurately reflected the genetic characteristics and TME of the patients, which could help guide clinical therapies for lung cancer patients with underlying diseases 112. The latest study described a method to create embedded bioprinting-enabled arrayed CPDOs utilizing embedded bioprinting technology 113. The model faithfully reproduced key attributes of TME, including elevated matrix stiffness and hypoxic conditions found in colorectal cancer 113.

To help overcome the limitations of existing models, it is necessary to further optimize the establishment protocol of CPDOs. With the continuous progress of technology, CPDOs will have a broader development prospect in cancer research and treatment.

## 3. Application of CPDOs for the study of NPs

**Figure 2:**
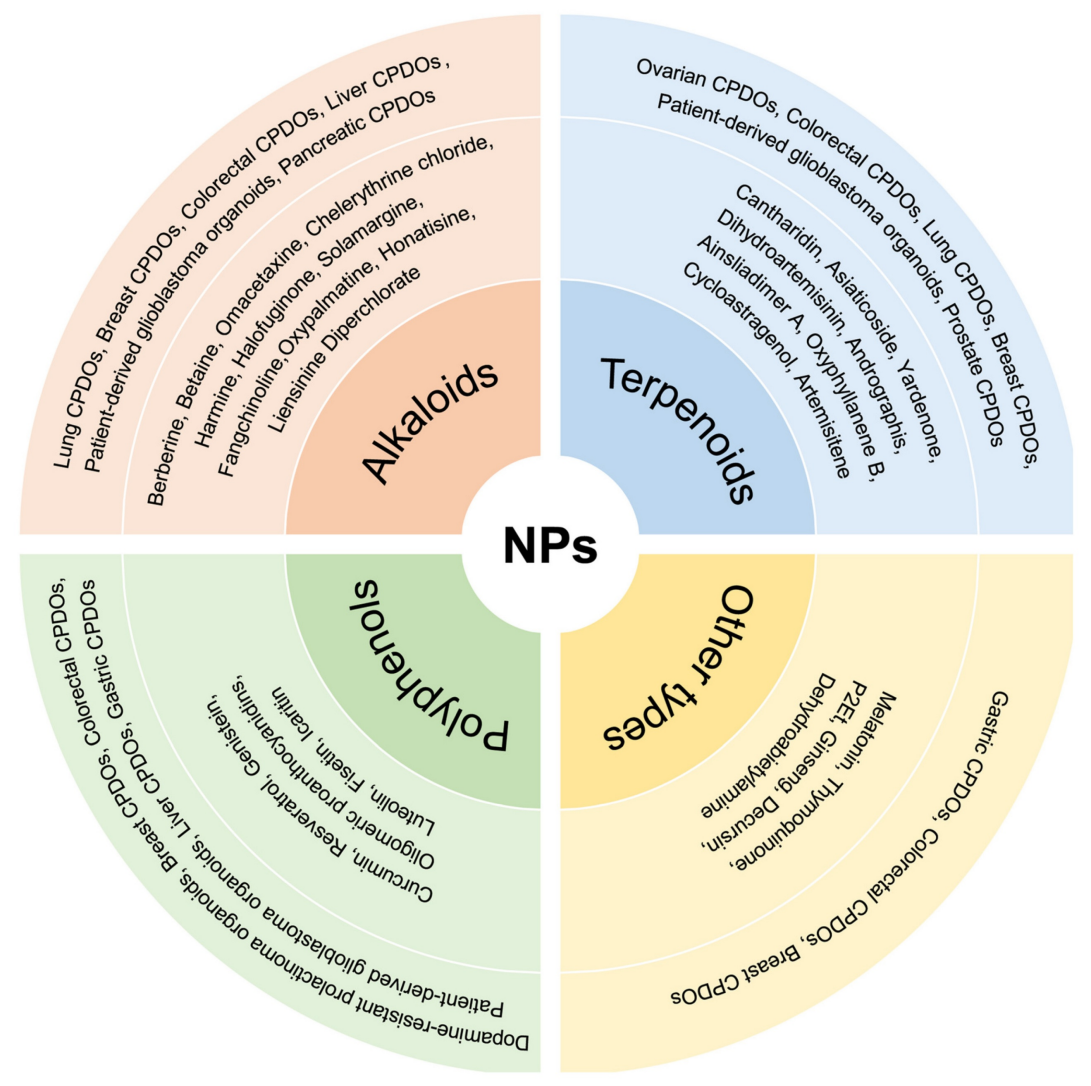
Application of different CPDOs for the study of NPs. Different kinds of CPDOs have been constructed for the study of NPs, including lung CPDOs, breast CPDOs, colorectal CPDOs, prostate CPDOs, and gastric CPDOs. The NPs involved are mainly categorized into alkaloids, polyphenols, terpenoids, and other types of compounds.

CPDOs faithfully preserve the molecular and cellular heterogeneity of primary tumors while closely recapitulating the histopathological features of patient tumors. These characteristics establish CPDOs as robust preclinical models for drug efficacy assessment, high-throughput screening, and mechanistic investigations, thereby facilitating the translation of potential therapeutic candidates into clinical applications. While numerous studies have employed CPDOs to evaluate conventional Western medicines 10, research investigating NPs remains comparatively limited (Figure 2). Nevertheless, existing evidence strongly suggests that NPs exhibit significant therapeutic potential across multiple cancer types when tested in CPDOs.

### 3.1 Alkaloids

#### 3.1.1 Berberine

Berberine is a natural isoquinoline alkaloid, which is mainly extracted from the roots and stems of various medicinal plants, such as the *Ranunculaceae*, *Rutaceae*, and *Berberidaceae* families 114. In lung cancer, berberine has been demonstrated to exert its anticancer activity by modulating tumor cell apoptosis, autophagy, metastasis, angiogenesis, immune responses, and chemotherapeutic responsiveness 115. A research paper in 2020 by **Li** *et al.* demonstrated that berberine could target epidermal growth factor receptor (EGFR) and suppress the growth of cancer cells by inhibiting EGFR activation 116. Interestingly, the authors observed that non-small cell lung CPDOs had an obvious sensitivity to berberine while cell lines showed resistance to it. This was because lung cancer cell lines might be different from lung CPDOs in terms of drug-gene associations and genotypes 116.

In 2022, a review by Jiang *et al.* illustrated that berberine could prevent the progression of colorectal cancer by regulating gene expression, the inflammatory response, oxidative stress, and so on 117. In addition, a study in 2022 by Okuno *et al.* reported that the combination treatment with two natural compounds, berberine and oligomeric proanthocyanidins (OPCs), markedly inhibited the growth of colorectal CPDOs 118. Furthermore, the study also revealed that the combination could exert synergistic anticancer effects of berberine and OPCs in CPDOs through enhancing cellular apoptosis and reducing the level of MYB *via* the PI3K-Akt signaling pathway. These data further supported the cell experimental results and offered important evidence for the combination of berberine and OPCs in the clinical therapy of colorectal cancer patients 118.

Studies have revealed that berberine possesses significant chemosensitizing and chemoprotective properties as a clinical adjunct agent, enhancing chemosensitivity and reversing chemotherapeutic drug resistance in many types of cancer 119. A study in 2022 by Okuno *et al.* reported that pancreatic CPDOs were generated to assess the effect of berberine to enhance the chemosensitivity of gemcitabine 120. Moreover, it was confirmed that berberine markedly reduced the number and size of CPDOs. The combination of berberine and gemcitabine exhibited a more pronounced anti-tumor effect in CPDOs. The data successfully verified the cell culture-based findings, suggesting that berberine significantly potentiated the anticancer potential of gemcitabine 120.

#### 3.1.2 Betaine

Betaine is also known as trimethylglycine and has beneficial biological effects in various human diseases. It was first identified in the 19th century in beets (*Beta vulgaris*), and it is distributed widely in animals, plants, and microorganisms 121. A study in 2020 by Li *et al.* found that non-small cell lung CPDOs and cell lines were resistant to betaine and there was no significant difference between the two models in the IC50 value of betaine 116.

#### 3.1.3 Omacetaxine

Omacetaxine mepusuccinate (homoharringtonine) is a plant alkaloid with antitumor properties, originally found in herbal extracts from the Chinese plum yew, *Cephalotaxus* 122. In order to test the anticancer effect of omacetaxine, Li *et al.* successfully constructed a library of liver CPDOs in 2021 123. They confirmed that omacetaxine not only decreased proliferation and increased apoptosis in CPDOs but also inhibited global protein synthesis and reduced levels of specific short-half-life proteins in CPDOs. The study demonstrated the potential clinical usefulness of omacetaxine as a novel anticancer agent in hepatocellular carcinoma and underscored the potential clinical usefulness of CPDOs as an ideal preclinical model for drug discovery 123.

#### 3.1.4 Chelerythrine chloride

Chelerythrine chloride, a natural benzodiazepine alkaloid, is mainly present in numerous herbal plants. Relevant studies have substantiated that chelerythrine chloride has strong antitumor pharmacological and biological activity 124, 125. In 2020, Li and colleagues successfully established a living biobank of CPDOs derived from 10 non-small cell lung cancer (NSCLC) patients for high-throughput screening of NPs, and they found the CPDOs were sensitive to chelerythrine chloride 116. These results showed that chelerythrine chloride exerted the highest inhibitory effect on the CPDOs and cell lines among the 5 natural compounds and it had equivalent sensitivity in both CPDOs and cell lines. It could be concluded that chelerythrine chloride, which has the highest anticancer activity and the least toxicity, is a novel natural anticancer compound for healing lung cancer 116.

#### 3.1.5 Harmine

Harmine is a natural β-carboline alkaloid that was derived from multiple medicinal plants. It has been reported that harmine is able to exhibit remarkable antitumor activities in multiple types of cancer through diverse mechanisms 126, 127. In the same study, Li *et al.* also observed that harmine significantly inhibited cell viability of CPDOs, but not NSCLC cell lines. These results played important roles in the introduction of these natural compounds into the personalized therapy of enrolled NSCLC patients 116.

#### 3.1.6 Halofuginone

Halofuginone, a natural alkaloid and an active derivative of febrifugine, is extracted from the Chinese herb *Dichroa febrifuga*. Plenty of evidence suggests that halofuginone possesses excellent anti-cancer, anti-fibrosis, and other properties 128, 129. The research of Li *et al.* in 2021 illustrated that halofuginone suppressed the cisplatin-resistant cells by the dual regulation of PI3K/AKT and MAPK signaling pathways 130. Then, they constructed two cisplatin-resistant lung CPDOs to further validate the anticancer effect of halofuginone and found that the inhibitory effect of halofuginone in cisplatin-resistant lung CPDOs was similar to that of lung cancer cell lines. Therefore, halofuginone can act as a promising cisplatin sensitizer to improve the prognosis of patients with cisplatin-resistant lung cancer in future clinical practice 130.

#### 3.1.7 Solamargine

Solamargine, an alkaloid natural compound isolated from a traditional Chinese herb called *Solanum nigrum L.*, has been widely applied to treat various diseases such as cancers, inflammation, and warts 131, 132. In 2022, Han *et al.* succeeded in establishing CPDOs derived from cisplatin-resistant lung cancer patients. Through high-throughput screening of natural product libraries, an alkaloid natural product solamargine was determined as a potential cisplatin sensitizer and therapeutic agent, which might offer a novel approach for further treating patients with advanced cisplatin-resistant lung cancer 133. Besides, it was proved that solamargine could exert its antitumor properties by inhibiting the hedgehog pathway and showed the synergistic inhibitory effect with cisplatin in cisplatin-resistant lung cancer cell lines 133.

#### 3.1.8 Fangchinoline

Fangchinoline, a bisbenzylisoquinoline alkaloid derived from the root of *Stephania tetrandra S.*, has been found to exhibit extensive pharmacological effects including anti-oxidant, anti-inflammatory, anticancer, and neural protection effects 134. The progression and metastasis of lung cancer are closely related to EGFR mutations 135. In a study in 2022 by Chen *et al.*, they collected tumor tissues from lung adenocarcinoma patients with EGFR mutation and wild-type EGFR to culture CPDOs according to the standard protocol 136. The CPDOs were then treated with different concentrations of fangchinoline for one week, and the drug was found to inhibit dose-dependently the growth of CPDOs, with more prominent inhibition in EGFR-mutant organoids 136.

#### 3.1.9 Oxypalmatine

Oxypalmatine is an active protoberberine-type alkaloid isolated from the bark of *Phellodendron amurense* (*Rutaceae*) 137. In 2023, Lin *et al.* successfully established breast CPDOs from tumor tissues characterized as luminal A, HER2-overexpressing, and triple-negative, and they used these CPDOs to evaluate the clinical value of oxypalmatine 35. They observed that oxypalmatine could effectively attenuate the growth of CPDOs and further elucidated the specific mechanism of oxypalmatine inhibiting the proliferation and inducing apoptosis of breast cancer cells. This suggested that oxypalmatine is a promising medicine, highlighting the clinical transformation of oxypalmatine in breast cancer treatment 35.

#### 3.1.10 Liensinine Diperchlorate

Liensinine perchlorate (LIN) is a natural alkaloid derived from the seed embryo of *Nelumbo nucifera Gaertn* and has superior anti-colorectal cancer activity 138. In another investigation by Lin *et al.*, they evaluated the synergistic inhibitory effect of the combination of natural compounds LIN and artemisitene in breast CPDOs 32. They observed that LIN and artemisitene reduced the growth of breast CPDOs in a dose-dependent manner, and confirmed that LIN could synergistically suppress the growth of breast CPDOs without obvious side effects. This preclinical data suggested that the combination of LIN and artemisitene is a promising regimen for breast cancer therapy which may improve the prognosis of breast cancer patients and process mitigate breast cancer progression 32.

#### 3.1.11 Honatisine

Honatisine, a distinctive heptacyclic diterpenoid alkaloid separated from Delphinium honanense, has exhibited significant cytotoxic activity 139. In 2024, Li *et al.* generated CPDOs from tumor tissues of patients with recurrent glioblastoma to further assess the anti-glioma properties of honatisine 140. The results showed that honatisine treatment repressed the growth of CPDOs and induced apoptosis compared with the control treatment. These data strongly supported that honatisine has a promising therapeutic prospect in recurrent glioblastoma 140.

### 3.2 Terpenoids

#### 3.2.1 Dihydroartemisinin

Artemisinin is a natural sesquiterpene lactone, which was initially extracted and isolated from *Artemisia annua L.*, and dihydroartemisinin is one of the derivatives of artemisinin 141. Dihydroartemisinin is not only an effective clinical medicine for the treatment of malaria but also exhibits superior anticancer activity in a variety of cancers 142. A very recent study in 2024 confirmed the synergistic cytotoxic effects of dihydroartemisinin and cisplatin using lung CPDOs 143. Moreover, the researchers further validated *in vitro* and in vivo that dihydroartemisinin was able to enhance the sensitivity of lung cancer cells to cisplatin by upregulating ZIP14 expression and inducing ferroptosis, which will provide a potential strategy for overcoming chemoresistance 143.

#### 3.2.2 Andrographis

Andrographis, a principle active compound of the Chinese herbal medicine *Andrographis paniculate*, possesses various activities such as anti-inflammatory, anti-obesity, anti-cancer, and other activities 144. A research paper in 2020 by Sharma *et al.* confirmed that the combined therapy effectively inhibited the growth and formation of CPDOs compared with 5-fluorouracil and andrographis alone 145. Hence, andrographis could mediate chemosensitization in colorectal cancer and had the synergistic anti-cancer activity with 5-fluorouracil. This suggested that andrographis stands as a promising natural therapeutic agent that can present a safer and cheaper option for adjuvant therapy of conventional chemotherapeutic drugs 145. Similarly, Shimura *et al.* also demonstrated that andrographis and another natural compound OPCS, exerted their superior combined anti-cancer effects in cell lines, xenograft animal models and CPDOs. Nevertheless, in CPDOs, there were large differences in the inhibitory effect and gene expression of combined therapy, which could be explained by the inherent tumor heterogeneity between the organoids 146.

#### 3.2.3 Cantharidin

Cantharidin, a natural terpenoid separated from blister beetles, has been used extensively in traditional Chinese medicine to cure various types of cancer 147, 148. Also in Li *et al.*'s study, they proved that cantharidin had a moderate inhibitory effect on cell viability of CPDOs, yet observed that the IC50 value of cantharidin in CPDOs was significantly higher than that in cell lines. This suggested that the sensitivity of Cantharidin in the two models was different 116.

#### 3.2.4 Asiaticoside

Asiaticoside, a natural triterpenoid saponin, is the major active ingredients of *Centella asiatica (L.) Urb.* and possesses diverse pharmacological properties including antitumor, neuroprotective, and wound healing 149. In 2024, Guo *et al.* collected the cells from the ascites of ovarian cancer patients and used these cells to establish CPDOs to evaluate the cytotoxic effect of asiaticoside on natural killer (NK) cells against ovarian cancer cells. The models were used to mimic the TME of ovarian cancer and exhibited high levels of TGF-β 150. They also observed that asiaticoside pretreatment effectively enhanced the antitumor ability of NK cells against CPDOs in the presence of high TGF-β levels. This suggested that asiaticoside may be a promising candidate to augment current NK cell-based immunotherapy strategies for ovarian cancer patients 150.

#### 3.2.5 Cycloastragenol

Cycloastragenol, an effective bioactive molecule derived from *Astragalus membranaceus*, possesses anti-inflammatory, anti-aging, and anticancer activities 151. In 2022, Deng *et al.* found that cycloastragenol could promote the expression of MHC-I in CPDOs and enhance the killing ability of CD8^+^T cells 152. Meanwhile, the combination of cycloastragenol and the PD-1 antibody was more effective in inhibiting the growth of CPDOs 152.

#### 3.2.6 Artemisitene

Artemisitene, an endoperoxide closely related to the famous antimalaria drug artemisinin, was originally isolated from the herb *Artemisia annua L.* 153. It possesses a variety of activities such as anti-rheumatoid arthritis, anti-lung damage, anti-ulcerative colitis, and so on 33. Recently, Chen *et al.* investigated the therapeutic potential effect of artemisitene on breast cancer. In their work, CPDOs were established to assess the clinical therapeutic efficacy of artemisitene in breast cancer. They reported that artemisitene inhibited the growth of breast CPDOs with different pathological subtypes and exhibited an excellent safety profile in contrast to conventional chemotherapy drugs 31. Moreover, they further proved the underlying mechanism of artemisinin-induced breast cancer cell apoptosis based on experiments. These results suggested that artemisitene can be an effective agent candidate for clinical breast cancer treatment 31.

#### 3.2.7 Ainsliadimer A

Ainsliadimer A, a dimeric sesquiterpene lactone, which is isolated from *Ainsliaea macrocephala*, has anticancer and anti-inflammatory properties 154. In 2023, Lv *et al.* observed that ainsliadimer A suppressed tumor growth in mice and the growth of CPDOs 155. Besides, the research also elucidated the specific mechanism by which ainsliadimer A induced apoptosis in colorectal cancer cells 155.

#### 3.2.8 Oxyphyllanene B

Oxyphyllanene B is a certain type of sesquiterpene. As early as 2005, it was reported that sesquiterpenes might act as potential anticancer agents and reduce cancer growth 156. In a study by Cui *et al.*, patient-derived glioblastoma organoids were established from the resected tumor tissues without enzymatic dissociation into single cells in 3D collagen gel, to further elucidate the anti-tumor effect of oxyphyllanene B and its underlying mechanism 157. They confirmed that oxyphyllanene B induced apoptosis in temozolomide-resistant glioblastoma cells and CPDOs in a time- and dose-dependent manner 157.

#### 3.2.9 Yardenone

Yardenone, a natural triterpenoid, is isolated from the marine sponges belonging to the Axinella genus 158. A study by Dai *et al.* reported the ability of sodwanone and yardenone triterpenoids to suppress the activation of hypoxia-inducible factor-1 (HIF-1), suggesting the potential role of these compounds in HIF-1 inhibition 159. Besides, in a very recent study by Peng *et al.* in 2024, they further explored the effect of yardenone 2 in HIF-1α regulation and demonstrated that yardenone 2 played a significant role in hypoxia 160. It was shown that yardenone 2 inhibited cell proliferation in prostate CPDOs, and altered the morphology and conformation of these organoids. This suggested that yardenone 2 may act as a novel HIF-1α inhibitor, thus providing a promising therapeutic strategy for the treatment of prostate cancer 160.

### 3.3 Polyphenols

#### 3.3.1 Curcumin

Curcumin, the active ingredient of the rhizomes of *Curcuma longa*, exhibits distinctive anticancer properties in multiple types of cancer by suppressing a variety of cellular signaling pathways 161. In a research paper in 2023, Miyazaki *et al.* developed and cultured colorectal CPDOs to demonstrate the anti-tumor effects of two natural compounds, curcumin, andrographis, and their combination. It was shown that the combined treatment with curcumin and andrographis significantly reduced the number and mean size of CPDOs 162. Additionally, ferrostatin-1, an inhibitor of ferroptosis, reversed the anti-cancer synergistic effect of the combination in both cancer cells and CPDOs. This illustrated that the combination therapy may exert its superior anticancer effect *via* the activation of the ferroptosis pathway 162.

#### 3.3.2 Resveratrol

Resveratrol, a natural phytoalexin, has been widely used to treat various types of cancer 26. A recent study in 2022 demonstrated that resveratrol had a stronger inhibitory effect on different subtypes of advanced breast CPDOs compared with conventional anti-breast cancer drugs 163. Moreover, the research also revealed that STAT3 activation was closely related to the resveratrol sensitivity of CPDOs. These results strongly supported the higher efficacy and broader spectrum of resveratrol against CPDOs and emphasized the promising clinical usefulness of resveratrol in advanced breast cancer 163.

#### 3.3.3 Genistein

Genistein, an isoflavone present in soy, has been proven to have a broad spectrum of pharmacological property and positive therapeutic effect in various diseases including cancer, obesity, osteoporosis, and metabolic syndrome 164. In a very recent study by Cheng *et al.* in 2024, dopamine-resistant prolactinoma organoids were successfully established to conduct high-throughput drug screening of the efficacy of 180 small molecule compounds. They finally identified that genistein presented the most superior anticancer effect among all tested compounds 165. Moreover, further experiments confirmed that genistein inhibited significantly and dose-dependently the proliferation and promoted apoptosis in CPDOs. This study revealed the role of genistein and its potential for clinical application, providing an attractive therapeutic strategy for the treatment of prolactinomas 165.

#### 3.3.4 Luteolin

Luteolin is a natural flavonoid extensively present in different plants like vegetables, fruits, and medicinal herbs. It has been shown that luteolin exhibits a variety of biological effects through distinct mechanisms and has been applied to treat a variety of human malignancies, including gastric cancer 166. In a recent study in 2023, Hao *et al.* successfully constructed CPDOs from tumor tissues of patients with gastric cancer, examined the anti-tumor effect of luteolin in different CPDOs, and elucidated its potential mechanism by transcriptome profiling 167. The study showed that luteolin significantly decreased the cluster size of almost all CPDOs in a dose-dependent manner, but the sensitivity of each organoid to luteolin was different, which might have resulted from the high heterogeneity of gastric cancer tumors. These results indicated the considerable potential of CPDOs for preclinical drug discovery and personalized drug treatment 167.

A study in 2018 by Yi *et al.* investigated the influence of luteolin in glioblastoma cells, patient-derived glioma initiating cells, and CPDOs. They found that the proliferation of CPDOs was inhibited when treated with luteolin, showing its superior antitumor activity 168. Next, they demonstrated the value of luteolin in combination with olaparib and ionizing radiation, indicating that it could synergistically enhance the effect of radiation and anticancer agents. These results suggested that the anticancer effects of luteolin can be extrapolated to treat patients with glioblastoma 168.

#### 3.3.5 Fisetin

Fisetin, a naturally occurring flavonoid found widely in various vegetables and fruits, has been shown to have anticancer effects in multiple types of cancer 169. In 2023, Kim *et al.* demonstrated that fisetin inhibited the viability of colorectal CPDOs in a dose-dependent manner 170. Next, they evaluated fisetin-induced tumor growth and examined the gene expression of tumor tissues in a colorectal cancer patient-derived organoid xenograft (PDOX) model 170. These findings suggested that fisetin is a potential candidate for the treatment of colorectal cancer.

#### 3.3.6 Icaritin

Icaritin, a natural compound extracted from the Chinese herbal plant *Epimedium*, has recently gained increasing attention due to its superior anti-cancer property 171. In 2024, Kang *et al.* established CPDOs using cancer cells obtained from patients with intrahepatic cholangiocarcinoma to verify the therapeutic effect of icaritin 172. It was observed that the combination of icaritin and gemcitabine plus cisplatin significantly inhibited the proliferation of CPDOs and effectively suppressed cancer progression. The findings suggested a promising avenue for novel therapeutic interventions in intrahepatic cholangiocarcinoma and indicated the substantiated role of icaritin in this specific patient population 172.

#### 3.3.7 Oligomeric proanthocyanidins

A group of proanthocyanidins present in grape seed extract, the shorter oligomers of which are called oligomeric proanthocyanidins (OPCs), has been shown the anti-colorectal cancer effect 173. In 2018, Toden and colleagues collected cancer cells from patients to generate colorectal CPDOs and evaluated the effectiveness of OPCs in CPDOs. Consistent with findings in cancer cell lines and mice xenografts, OPCs consistently suppressed the formation and growth of CPDOs and regulated the expression of cell cycle-associated genes 174, 175. These data highlighted the promising use of OPCs as a chemopreventive agent in colorectal cancer, with great clinical therapeutic potential.

### 3.4 Other kinds of compounds

#### 3.4.1 Hormone

Melatonin, a natural amine hormone, is synthesized in the pineal gland of mammals and humans exclusively at night 176. It has been identified that melatonin possesses a wide range of bioactive effects, such as sleep-wake cycle control, antioxidant, anti-inflammation, anticancer, and so forth 177. In a study by Zhao *et al.* in 2022, CPDOs were generated from colorectal cancer patients for the purpose of evaluating the synergistic anticancer effects of melatonin and andrographis 178. The findings confirmed that the combination of melatonin and andrographis exhibited more remarkable anticancer effect in cancer cells, xenograft animal models, and CPDOs compared with the individual compounds. This provided a potential therapeutic strategy for colorectal cancer 178.

#### 3.4.2 Quinones

Thymoquinone, the principle bioactive constituent of *Nigella sativa* seeds, has been proven to have potent therapeutic properties *in vivo* and *in vitro* models, especially in cancer, where thymoquinone could effectively combat diverse human cancers by modulating different signaling pathways 179. In 2022, Bitar *et al.* successfully established CPDOs using tumor samples from colorectal cancer patients with different clinical manifestations and investigated the radiosensitizing effect of thymoquinone. Thymoquinone could radiosensitize cancer stem cells by decreasing both the count and size of CPDOs and suppress stemness and DNA repair mechanisms 180. Interestingly, they observed that thymoquinone, radiation, and the combination treatments showed different responses in three CPDOs, probably due to differences in their clinical and histopathological characteristics 180.

#### 3.4.3 Coumarins

Decursin is a coumarin extracted from the roots of the medicinal plant *Angelica gigas*. A very recent review in 2024, systematically summarized that decursin has an effective therapeutic role in cancers, which is considered as a promising cancer therapeutic agent because of its potent anticancer activity 181. A study in 2021 by Kim *et al.* revealed that decursin reduced the growth of spheroids and CPDOs. Consistent with *in vivo* and *in vitro* results, decursin inhibited autophagic flux and decreased the expression of lysosomal protein cathepsin C in CPDOs 182. This study used gastric CPDOs to further verify the anticancer effects of decursin, enhancing the clinical relevance of *in vitro* findings 182.

#### 3.4.4 Amines

Dehydroabietylamine, also known as leelamine, a natural compound extracted from pine bark, exhibited the antitumor activity in the treatment of many types of cancer 183. In a very recent study by Ma *et al.* in 2024, CPDOs were successfully constructed and cultured from tumor tissues of patients with gastric cancer to evaluate the inhibitory effect of dehydroabietylamine 184. They discovered that dehydroabietylamine decreased the viability and suppressed the proliferation of CPDOs, showing the significant dose-dependent effect. These data illustrated the effect of dehydroabietylamine and its potential for clinical application, providing potential drug candidates for the treatment of gastric cancer 184.

### 3.5 Extract of NPs

#### 3.5.1 Ginseng

Ginseng is one of the most valuable and common Chinese medicines and has been used and researched not only in ancient China but also worldwide. Ginseng and its major extracts could significantly inhibit the development of colorectal cancer by different mechanisms 185. A recent study by Okuno *et al.* in 2023, they used CPDOs to validate the anticancer activity of ginseng extract. The finding suggested that ginseng inhibited the growth and formation of CPDOs and significantly downregulated the expression of DNMTs in CPDOs 186. These experimental data showed the anticancer potential of Ginseng in colorectal cancer and laid the groundwork for its clinical application in therapy.

#### 3.5.2 P2Et

A polyphenol-rich extract of *Caesalpinia spinosa* (P2Et) has been reported to possess a tumor-killing effect and to regulate the specific immune response in both breast cancer and melanoma 187. In a study in 2020, Urueña *et al.* observed that P2Et had significant cytotoxicity to breast CPDOs, and the tumor-killing effect was more apparent in combination with standard chemotherapy. This suggested that P2Et can be used as a favorable co‑adjuvant to improve the chemotherapy strategy of antitumor therapy in breast cancer patients 188.

## 4. Challenges and Future Perspectives

**Figure 3:**
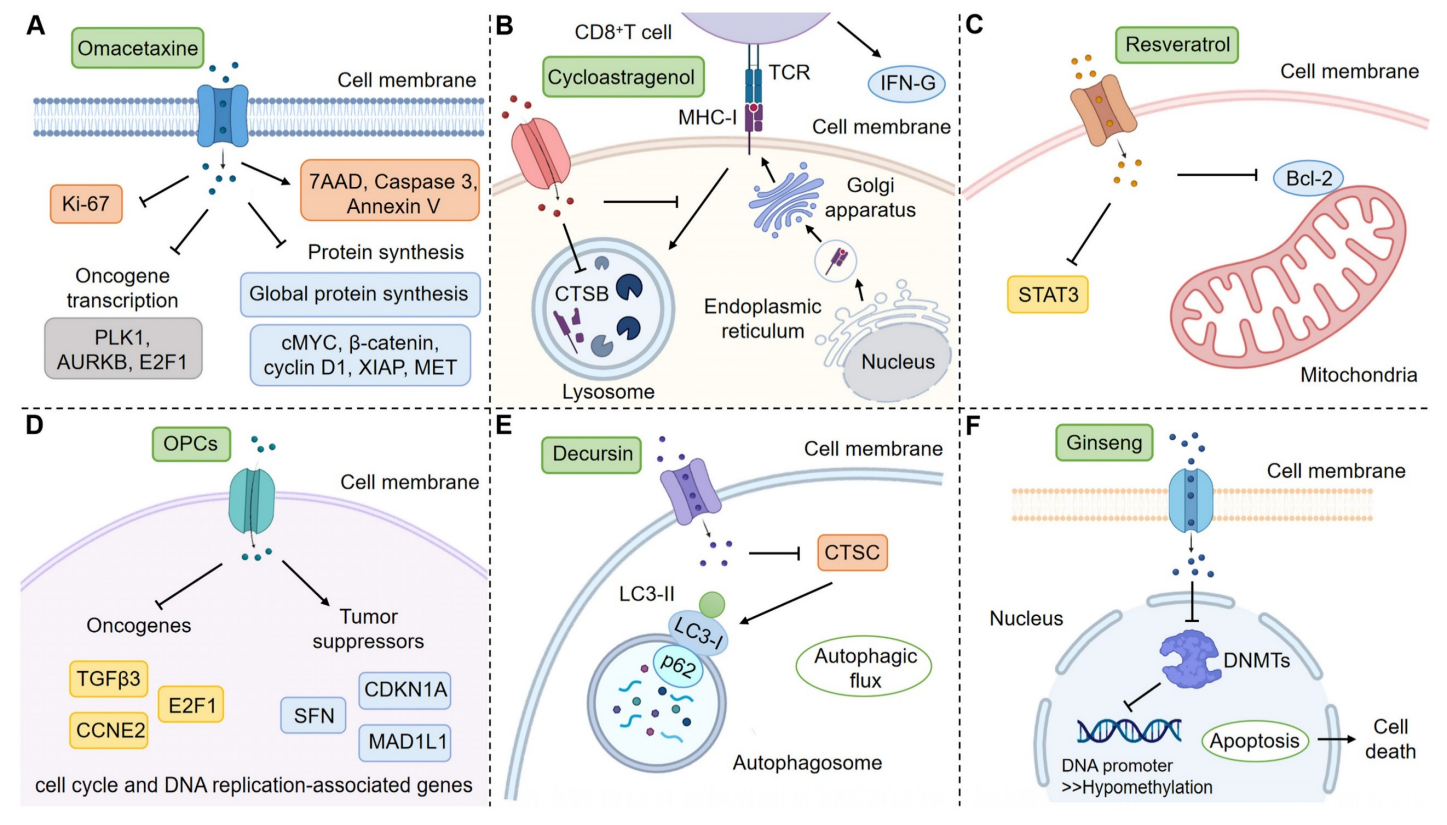
** Mechanisms of action of some NPs in CPDOs.** CPDOs can be applied to mechanism studies of NPs. (A) Liver CPDOs are applied to explore the mechanism of Omacetaxine. (B) Colorectal CPDOs are applied to explore the mechanism of Cycloastragenol. (C) Breast CPDOs are applied to explore the mechanism of Resveratrol. (D) Colorectal CPDOs are applied to explore the mechanism of OPCs. (E) Gastric CPDOs are applied to explore the mechanism of Decursin. (F) Colorectal CPDOs are applied to explore the mechanism of Ginseng. This figure was created with the help of BioRender (https://www.biorender.com/).

In this work, we systematically summarized the application of CPDOs for the study of NPs. However, most current studies focused on the evaluation of drug efficacy, and there were fewer reports on the mechanism study. These studies on mechanisms simply detected the changes in relevant genes and proteins and did not conduct more in-depth studies (Figure 3) 123, 152, 163, 175, 182, 186. We hope that researchers will be able to utilize CPDOs to further explore specific mechanisms of NPs, for example, gene-deficient CPDOs can be considered 189.

Although CPDOs have significant advantages in simulating human organs and screening anticancer drugs compared with cellular and animal models 190, they inevitably have certain limitations. First of all, different patients have different clinical manifestations and pathological subtypes, so there may be some differences between CPDOs established from the tumor tissues of these patients. As a result, CPDOs exhibit diverse responses when treated with NPs 136, 146, 163, 167, 180, that lead to the inability of CPDOs to accurately evaluate the efficacy and mechanism of NPs.

We hope to reach an expert consensus that can define a comprehensive quality control guideline of CPDOs and establish a standardized cultivation protocol of CPDOs. This will enable subsequent researchers to better utilize CPDOs to study the efficacy of NPs. We propose the following suggestions. First, the source of patients' tumor tissues used to establish CPDOs should be clear. Second, the clinical manifestations and pathological subtypes of different cancer patients should be strictly distinguished. Moreover, a screening criterion for the inclusion of patient tumor tissues in the establishment of CPDOs should be determined, in order to establish a more standardized culture protocol of CPDOs. Such a criterion should take into account the cancer stages and clinical manifestations of patients, as well as their genomes. Besides, a standardized operational guideline should be determined, which includes methods of human tissues obtained from cancer patients by professional doctors and ways of long-term preservation and transportation of the tumor tissues. Additionally, an identification method should also be proposed to judge the success of CPDOs, such as the validation of biomarkers and assessment of genomic stability.

Furthermore, there are some translational gaps between current CPDO-based NP studies and clinical trials. This phenomenon is mainly due to factors such as individual differences in patients, differences in drug pharmacokinetics *in vivo*, and tumor specificity. For example, pharmacokinetic barriers of NPs in CPDOs are yet to be addressed. The application of suspension culture and agitation can increase the scalability of CPDOs, which will facilitate rapid, personalized, and tumor type-agnostic drug testing in a clinically relevant timeframe 99. Currently, a variety of CPDO biobanks have been established, including colorectal cancer 52, gastric cancer 191, and kidney cancer 192. These CPDO biobanks can precisely predict the responses to different therapeutic drugs, providing the guidance for drug selection and drug combination therapy for clinical cancer patients. As a further example, it is difficult to exactly match the drug dose settings in CPDOs with those used in clinical applications. At present, PDOX models have been developed to evaluate the performance of NPs in the *in vivo* environments 170. However, the results obtained from PDOX models and CPDOs are not completely consistent, which may be attributable to the underdose and biological differences between two systems 10. The dose-conversion relationship of NPs between CPDOs and clinical trials should be confirmed, but this needs to be studied and validated experimentally.

Despite the above challenges, more and more studies have addressed these issues by combining CPDOs with other cells and techniques. CPDOs can be co-cultured with CAFs 69, 105, 106, T lymphocytes 69, 107, PBL 104, MDSCs 107, genetically-engineered cells 81, and other cells, for the study of immune interactions of drugs in cancer. The effects of clinically used anticancer drugs have been demonstrated in co-culture models 105. Unfortunately, these methods have not yet been applied to the study of NPs. Moreover, microwell array chips 41, microfluidic technology 109-111, 3D bioprinting 112, 113, and network-based machine learning 193 have made some progress in the application of CPDOs in drug research. CPDOs combined with artificial intelligence can accurately predict and analyze the therapeutic effects of anticancer drugs, thereby enhancing drug safety and optimizing the personalized clinical treatment strategy of cancer patients 194.

The study of NPs in these CPDOs has a promising future, despite several challenges such as technical and analytical difficulties. Most importantly, further evidence, particularly those in clinical trials, is required to substantiate the utility of CPDOs in cancer.

## 5. Conclusions

In summary, CPDOs demonstrate high fidelity in predicting drug sensitivity profiles of primary tumors, establishing themselves as robust preclinical platforms for drug efficacy evaluation and high-throughput screening. With the improvement of cancer organoid technologies, CPDOs will become increasingly valuable for assessing the therapeutic potential and safety profiles of NPs, further validating their promising roles in cancer treatment strategies.

## Figures and Tables

**Table 1:** Identification of markers for representative CPDOs.

Cancer types	CPDOs types	Sample source	Identification of markers	References
Lung cancer	Lung adenocarcinoma	Surgically resected tumor tissues, pleural effusion	TTF-1	37
		Surgically resected tumor tissues, biopsied tumor samples		38
		Surgical specimens, bronchoscopy biopsies, pleural effusion, blood-circulating tumor cells, sputum samples		39
		Surgically resected tumor tissues	TTF-1, CK7	40
		Collected lung cancer samples		41
		Surgically resected tumor tissues	TTF-1, CK7, napsin A	42, 43
		Mainly malignant serous effusion		44
	Lung squamous cell carcinoma	Surgical specimens, bronchoscopy biopsies, pleural effusion, sputum samples	p40	39
		Surgically resected tumor tissues	CK5/6, p63	40, 42
		Surgically resected tumor tissues, biopsied tumor samples		38
		Collected lung cancer samples	CK5/6, p40	41
		Surgically resected tumor tissues	CK5, p40	43
		Mainly malignant serous effusion	CK5/6, p40, p63	44
	Adenosquamous carcinoma	Surgically resected tumor tissues	CK7, CK5/6, p63	42
	Large cell neuroendocrine carcinoma	Surgical specimens, bronchoscopy biopsies, pleural effusion	NCAM 1	39
		Surgically resected tumor tissues	CK7, CD133	42
	Small cell lung cancer	Surgical specimens, bronchoscopy biopsies, pleural effusion, blood-circulating tumor cells, sputum samples	NCAM 1	39
		Biopsied tumor samples	CD56, synaptophysin, chromogranin	45
		Surgically resected tumor tissues	CD56, synaptophysin, TTF-1	42
		Mainly malignant serous effusion	CD56, synaptophysin, TTF-1, hromogranin A	44
Breast cancer	All major disease subtypes	Surgically resected tumor tissues	ER, PR, HER2	46-48
Gastrointestina cancer	CPDOs from metastatic gastrointestinal cancers	Biopsied tumor samples	CDX2、CK7	49
Rectal cancer	Rectal cancer	Surgically resected tumor tissues, biopsied tumor samples	CDX2, β-catenin, CK20, MUC2, E-cadherin	50
	Locally advanced rectal cancer	Biopsied tumor samples	CDX2, β-catenin, CK20, CK-pan	51
Colorectal cancer	CPDOs from colorectal cancer with paired liver metastasis	Surgically resected tumor tissues		52
	Primary or metastatic colorectal cancer	Surgically resected tumor tissues	MUC2, p53	53
Prostate cancer	Prostate cancer	Surgically resected tumor tissues	PSA, NKX3.1, AR, CK8, p63, CK5	54
		Surgically resected tumor tissues, biopsied tumor samples	PSA, AR, CK8, CK18, Probasin, p63, CK5	55
	Castration-resistant prostate cancer adenocarcinoma	Biopsied tumor samples	AR, KLK3, ENO2 (NSE), NKX3.1, AR-V7, CHGA, SYP, PSMA, HOXB13	56
Gastric cancer	Gastric cancer	Surgically resected tumor tissues	CDX2	57
		Surgically resected tumor tissues	CEA, CK7	58
		Surgically resected tumor tissues	CEA, CK7, CDH17	59
		Biopsied tumor samples		60
Liver cancer	Hepatocellular carcinoma	Surgically resected tumor tissues	AFP, HepPar 1	61, 62
			GPC3, β-catenin	63
			AFP, GPC3	64
		Biopsied tumor samples	AFP, GPC3, GS, HSP70, KRT7, KRT19	65
	Intrahepatic cholangiocarcinoma	Surgically resected tumor tissues	KRT19, EpCAM	62
			CK19, EpCAM	63
	Cholangiocarcinoma	Surgically resected tumor tissues	EpCAM	61
	Hepatoblastoma	Surgically resected tumor tissues	AFP, GPC3, CK19, EpCAM	63
	Combined hepatocellular-cholangiocarcinoma	Surgically resected tumor tissues	AFP, HepPar 1, EpCAM	61
			AFP, EpCAM	62
			AFP, GPC3, CK19, EpCAM	63
Biliary tract carcinomas	Intrahepatic cholangiocarcinoma, pancreatic ductal adenocarcinoma, gallbladder cancer	Surgically resected tumor tissues	CK7, MUC1, EpCAM	66
Pancreatic cancer	Pancreatic ductal adenocarcinoma	Surgically resected tumor tissues	CK19, Laminin α5	67
		Biopsied tumor samples	CK19, E-cadherin	68
		Surgical specimens, rapid autopsy specimens, ascites	PDX1, CK19	69
		Biopsied tumor samples	GATA6, KRT5/6, KRT17, p63	70
		Surgically resected tumor tissues	KRT19, GATA6, SOX9	71
Kidney cancer	Renal cell carcinoma	Surgically resected tumor tissues	AMACR, CK7, vimentin, CD10, PAX2, CK8/18, E-cadherin	72
	Clear cell renal cell cancer	Surgically resected tumor tissues	CXCR4, MET, CD44, VCAM1	73
			SOX2, CK8/18, HIF1 α, E-cadherin	74
Ovarian cancer	Mainly high-grade serous ovarian cancer	Biopsied tumor samples	PAX8, CK7, Erα, PR, CK8/18, E-cadherin	75
	Ovarian cancer	EpCAM+ cancer cells from ovarian cancer patient ascites	CK8	76
	High-grade serous ovarian cancer	Tumor samples from surgeries, laparoscopic biopsies or ascites paracentesis	PAX8, CK7, WT1	77
	Ovarian cancer	Surgically resected tumor tissues	PAX8, p53	78
	High-grade serous ovarian cancer			79
Glioblastoma	Glioblastoma	Collected tumor tissues	GFAP, S100B, DCX, NESTIN, BLBP, HOPX, SOX2, OLIG2, IBA1	80, 81

## Abbreviations

3D: three-dimensional
ADMET: absorption, distribution, metabolism, excretion and toxicity
AFP: alpha-fetoprotein
ALI: air-liquid interface
AMACR: alpha-methylacyl-CoA racemase
AO: airway organoid
AR: androgen receptor
AR-V7: androgen receptor variant 7
BLBP: brain lipid-binding protein
CAFs: cancer-associated fibroblasts
CAR: chimeric antigen receptor
CD: cluster of differentiation
CDH17: cadherin 17
CDX2: caudal type homeobox 2
CEA: carcinoembryonic antigen
CHGA: chromogranin A
CK: cytokeratin
CK-pan: pan-cytokeratin
CPDOs: cancer patient-derived organoids
CXCR4: C-X-C chemokine receptor 4
DCX: doublecortin
EGF: epidermal growth factor
EGFR: epidermal growth factor receptor
ENO2: enolase 2
EpCAM: epithelial cell adhesion molecule
ER: estrogen receptor
Erα: estrogen receptor alpha
FGF: fibroblast growth factor
GATA6: GATA binding protein 6
GFAP: glial fibrillary acidic protein
GPC3: glypican 3
GS: glutamine synthetase
HER2: human epidermal growth factor receptor 2
HGF: hepatocyte growth factor
HIF: hypoxia-inducible factor
HOPX: HOP homeobox
HOXB13: homeobox B13
HSP70: heat shock protein 70
IARC: International Agency for Research on Cancer
IBA1: allograft inflammatory factor 1
KLK3: kallikrein related peptidase 3
KRT: keratin
LIN: liensinine perchlorate
MAPK: mitogen-activated protein kinase
MDSCs: myeloid-derived suppressor cells
MET: mesenchymal-epithelial transition factor
MUC: mucin
NK: natural killer
NKX3.1: NK3 homeobox 1
NPs: natural products
NSCLC: non-small cells lung cancer
OLIG2: oligodendrocyte transcription factor 2
OPCs: oligomeric proanthocyanidins
p38: protein 38
p40: protein 40
p53: protein 53
p63: protein 63
PAX2: paired box 2
PAX8: paired box 8
PBL: peripheral blood lymphocytes
PD-1: programmed cell death protein 1
PDCs: patient-derived cancer cells
PDOX: patient-derived organoid xenograft
PDX1: pancreatic duodenal homeobox 1
PDXs: patient-derived xenografts
PI3K/AKT: phosphatidylinositol 3-kinase/protein kinase B
PR: progesterone receptor
PSA: prostate-specific antigen
PSCs: pancreatic stellate cells
PSMA: prostate-specific membrane antigen
S100B: S100 calcium binding protein B
SOX: SRY-box transcription factor
STAT3: signal transducer and activator of transcription 3
SYP: synaptophysin
TGF-β: transforming growth factor-beta
TME: tumor microenvironment
TP53: tumor protein 53
TTF-1: thyroid transcription factor-1
VCAM1: vascular cell adhesion molecule 1
WT1: wilms' tumor 1
ZIP14: ZRT/IRT-like protein 14

## References

[1] Bray F, Laversanne M, Sung H, Ferlay J, Siegel RL, Soerjomataram I (2024). Global cancer statistics 2022: GLOBOCAN estimates of incidence and mortality worldwide for 36 cancers in 185 countries. CA Cancer J Clin.

[2] Liao L (2025). Inequality in breast cancer: Global statistics from 2022 to 2050. Breast.

[3] Rossi G, Manfrin A, Lutolf MP (2018). Progress and potential in organoid research. Nat Rev Genet.

[4] Xu H, Jiao D, Liu A, Wu K (2022). Tumor organoids: applications in cancer modeling and potentials in precision medicine. J Hematol Oncol.

[5] Qu J, Kalyani FS, Liu L, Cheng T, Chen L (2021). Tumor organoids: synergistic applications, current challenges, and future prospects in cancer therapy. Cancer Commun.

[6] Sato T, Stange DE, Ferrante M, Vries RGJ, van Es JH, van den Brink S (2011). Long-term Expansion of Epithelial Organoids From Human Colon, Adenoma, Adenocarcinoma, and Barrett's Epithelium. Gastroenterology.

[7] Driehuis E, Kretzschmar K, Clevers H (2020). Establishment of patient-derived cancer organoids for drug-screening applications. Nat Protoc.

[8] Lv J, Du X, Wang M, Su J, Wei Y, Xu C (2024). Construction of tumor organoids and their application to cancer research and therapy. Theranostics.

[9] Lo Y-H, Karlsson K, Kuo CJ (2020). Applications of organoids for cancer biology and precision medicine. Nat Cancer.

[10] Mao Y, Wang W, Yang J, Zhou X, Lu Y, Gao J (2024). Drug repurposing screening and mechanism analysis based on human colorectal cancer organoids. Protein Cell.

[11] Li Z, Xu H, Gong Y, Chen W, Zhan Y, Yu L (2021). Patient-Derived Upper Tract Urothelial Carcinoma Organoids as a Platform for Drug Screening. Adv Sci.

[12] Chen Y, Su L, Huang C, Wu S, Qiu X, Zhao X (2021). Galactosyltransferase B4GALT1 confers chemoresistance in pancreatic ductal adenocarcinomas by upregulating N-linked glycosylation of CDK11(p110). Cancer Lett.

[13] Su L, Chen Y, Huang C, Wu S, Wang X, Zhao X (2023). Targeting Src reactivates pyroptosis to reverse chemoresistance in lung and pancreatic cancer models. Sci Transl Med.

[14] Love JR, Karthaus WR (2024). Next-Generation Modeling of Cancer Using Organoids. Cold Spring Harb Perspect Med.

[15] Christin JR, Shen MM (2022). Modeling tumor plasticity in organoid models of human cancer. Trends Cancer.

[16] Yang YH, Mao JW, Tan XL (2020). Research progress on the source, production, and anti-cancer mechanisms of paclitaxel. Chin J Nat Med.

[17] Zhao S, Tang Y, Wang R, Najafi M (2022). Mechanisms of cancer cell death induction by paclitaxel: an updated review. Apoptosis.

[18] Chen Q, Xu S, Liu S, Wang Y, Liu G (2022). Emerging nanomedicines of paclitaxel for cancer treatment. J Control Release.

[19] Khaiwa N, Maarouf NR, Darwish MH, Alhamad DWM, Sebastian A, Hamad M (2021). Camptothecin's journey from discovery to WHO Essential Medicine: Fifty years of promise. Eur J Med Chem.

[20] Wang X, Zhuang Y, Wang Y, Jiang M, Yao L (2023). The recent developments of camptothecin and its derivatives as potential anti-tumor agents. Eur J Med Chem.

[21] Liu Z, Yuan Y, Wang N, Yu P, Teng Y (2024). Drug combinations of camptothecin derivatives promote the antitumor properties. Eur J Med Chem.

[22] Clark I, Brougham MFH, Spears N, Mitchell RT (2023). The impact of vincristine on testicular development and function in childhood cancer. Hum Reprod Update.

[23] Shukla R, Singh A, Singh KK (2024). Vincristine-based nanoformulations: a preclinical and clinical studies overview. Drug Deliv Transl Res.

[24] Weng W, Goel A (2022). Curcumin and colorectal cancer: An update and current perspective on this natural medicine. Semin Cancer Biol.

[25] Wang W, Li M, Wang L, Chen L, Goh BC (2023). Curcumin in cancer therapy: Exploring molecular mechanisms and overcoming clinical challenges. Cancer Lett.

[26] Ren B, Kwah MX, Liu C, Ma Z, Shanmugam MK, Ding L (2021). Resveratrol for cancer therapy: Challenges and future perspectives. Cancer Lett.

[27] Tang SM, Deng XT, Zhou J, Li QP, Ge XX, Miao L (2020). Pharmacological basis and new insights of quercetin action in respect to its anti-cancer effects. Biomed Pharmacother.

[28] Dai S, Liu Y, Zhao F, Wang H, Shao T, Xu Z (2022). Aqueous extract of Taxus chinensis var. mairei targeting CD47 enhanced antitumor effects in non-small cell lung cancer. Biomed Pharmacother.

[29] Mao JT, Xue B, Lu QY, Lundmark L, Burns W, Yang J (2023). Combinations of grape seed procyanidin extract and milk thistle silymarin extract against lung cancer - The role of MiR-663a and FHIT. Life Sci.

[30] Chen J, Huang X, Tao C, Xiao T, Li X, Zeng Q (2019). Artemether Attenuates the Progression of Non-small Cell Lung Cancer by Inducing Apoptosis, Cell Cycle Arrest and Promoting Cellular Senescence. Biol Pharm Bull.

[31] Chen D, Li G, Luo L, Lin T, Chu X, Liu K (2024). Artemisitene induces apoptosis of breast cancer cells by targeting FDFT1 and inhibits the growth of breast cancer patient-derived organoids. Phytomedicine.

[32] Lin X, Lin T, Liu M, Chen D, Chen J (2024). Liensinine diperchlorate and artemisitene synergistically attenuate breast cancer progression through suppressing PI3K-AKT signaling and their efficiency in breast cancer patient-derived organoids. Biomed Pharmacother.

[33] Lin X, Chen J (2023). Artemisitene: a promising natural drug candidate with various biological activities needs to confirm the interactional targets. Front Pharmacol.

[34] Lin X, Liu J, Zou Y, Tao C, Chen J (2022). Xanthotoxol suppresses non-small cell lung cancer progression and might improve patients' prognosis. Phytomedicine.

[35] Lin X, Chen D, Chu X, Luo L, Liu Z, Chen J (2023). Oxypalmatine regulates proliferation and apoptosis of breast cancer cells by inhibiting PI3K/AKT signaling and its efficacy against breast cancer organoids. Phytomedicine.

[36] Atanasov AG, Zotchev SB, Dirsch VM, Supuran CT (2021). Natural products in drug discovery: advances and opportunities. Nat Rev Drug Discovery.

[37] Kim S-Y, Kim S-M, Lim S, Lee JY, Choi S-J, Yang S-D (2021). Modeling Clinical Responses to Targeted Therapies by Patient-Derived Organoids of Advanced Lung Adenocarcinoma. Clin Cancer Res.

[38] Dijkstra KK, Monkhorst K, Schipper LJ, Hartemink KJ, Smit EF, Kaing S (2020). Challenges in Establishing Pure Lung Cancer Organoids Limit Their Utility for Personalized Medicine. Cell Rep.

[39] Ebisudani T, Hamamoto J, Togasaki K, Mitsuishi A, Sugihara K, Shinozaki T (2023). Genotype-phenotype mapping of a patient-derived lung cancer organoid biobank identifies NKX2-1-defined Wnt dependency in lung adenocarcinoma. Cell Rep.

[40] Shi R, Radulovich N, Ng C, Liu N, Notsuda H, Cabanero M (2020). Organoid Cultures as Preclinical Models of Non-Small Cell Lung Cancer. Clin Cancer Res.

[41] Hu Y, Sui X, Song F, Li Y, Li K, Chen Z (2021). Lung cancer organoids analyzed on microwell arrays predict drug responses of patients within a week. Nat Commun.

[42] Kim M, Mun H, Sung CO, Cho EJ, Jeon H-J, Chun S-M (2019). Patient-derived lung cancer organoids as in vitro cancer models for therapeutic screening. Nat Commun.

[43] Li Z, Qian Y, Li W, Liu L, Yu L, Liu X (2020). Human Lung Adenocarcinoma-Derived Organoid Models for Drug Screening. iScience.

[44] Wang H-M, Zhang C-Y, Peng K-C, Chen Z-X, Su J-W, Li Y-F (2023). Using patient-derived organoids to predict locally advanced or metastatic lung cancer tumor response: A real-world study. Cell Rep Med.

[45] Choi SY, Cho Y-H, Kim D-S, Ji W, Choi C-M, Lee JC (2021). Establishment and Long-Term Expansion of Small Cell Lung Cancer Patient-Derived Tumor Organoids. Int J Mol Sci.

[46] Sachs N, de Ligt J, Kopper O, Gogola E, Bounova G, Weeber F (2018). A Living Biobank of Breast Cancer Organoids Captures Disease Heterogeneity. Cell.

[47] Chen P, Zhang X, Ding R, Yang L, Lyu X, Zeng J (2021). Patient-Derived Organoids Can Guide Personalized-Therapies for Patients with Advanced Breast Cancer. Adv Sci.

[48] Dekkers JF, van Vliet EJ, Sachs N, Rosenbluth JM, Kopper O, Rebel HG (2021). Long-term culture, genetic manipulation and xenotransplantation of human normal and breast cancer organoids. Nat Protoc.

[49] Vlachogiannis G, Hedayat S, Vatsiou A, Jamin Y, Fernández-Mateos J, Khan K (2018). Patient-derived organoids model treatment response of metastatic gastrointestinal cancers. Science.

[50] Ganesh K, Wu C, O'Rourke KP, Szeglin BC, Zheng Y, Sauvé C-EG (2019). A rectal cancer organoid platform to study individual responses to chemoradiation. Nat Med.

[51] Yao Y, Xu X, Yang L, Zhu J, Wan J, Shen L (2020). Patient-Derived Organoids Predict Chemoradiation Responses of Locally Advanced Rectal Cancer. Cell Stem Cell.

[52] Mo S, Tang P, Luo W, Zhang L, Li Y, Hu X (2022). Patient-Derived Organoids from Colorectal Cancer with Paired Liver Metastasis Reveal Tumor Heterogeneity and Predict Response to Chemotherapy. Adv Sci.

[53] Tan T, Mouradov D, Lee M, Gard G, Hirokawa Y, Li S (2023). Unified framework for patient-derived, tumor-organoid-based predictive testing of standard-of-care therapies in metastatic colorectal cancer. Cell Rep Med.

[54] Karthaus Wouter R, Iaquinta Phillip J, Drost J, Gracanin A, van Boxtel R, Wongvipat J (2014). Identification of Multipotent Luminal Progenitor Cells in Human Prostate Organoid Cultures. Cell.

[55] Drost J, Karthaus WR, Gao D, Driehuis E, Sawyers CL, Chen Y (2016). Organoid culture systems for prostate epithelial and cancer tissue. Nat Protoc.

[56] Mosquera MJ, Kim S, Bareja R, Fang Z, Cai S, Pan H (2021). Extracellular Matrix in Synthetic Hydrogel-Based Prostate Cancer Organoids Regulate Therapeutic Response to EZH2 and DRD2 Inhibitors. Adv Mater.

[57] Zu M, Hao X, Ning J, Zhou X, Gong Y, Lang Y (2023). Patient-derived organoid culture of gastric cancer for disease modeling and drug sensitivity testing. Biomed Pharmacother.

[58] Zhao Y, Li S, Zhu L, Huang M, Xie Y, Song X (2024). Personalized drug screening using patient-derived organoid and its clinical relevance in gastric cancer. Cell Rep Med.

[59] Seidlitz T, Merker SR, Rothe A, Zakrzewski F, von Neubeck C, Grützmann K (2019). Human gastric cancer modelling using organoids. Gut.

[60] Schmäche T, Fohgrub J, Klimova A, Laaber K, Drukewitz S, Merboth F (2024). Stratifying esophago-gastric cancer treatment using a patient-derived organoid-based threshold. Mol Cancer.

[61] Broutier L, Mastrogiovanni G, Verstegen MMA, Francies HE, Gavarró LM, Bradshaw CR (2017). Human primary liver cancer-derived organoid cultures for disease modeling and drug screening. Nat Med.

[62] Yang H, Cheng J, Zhuang H, Xu H, Wang Y, Zhang T (2024). Pharmacogenomic profiling of intra-tumor heterogeneity using a large organoid biobank of liver cancer. Cancer Cell.

[63] Ji S, Feng L, Fu Z, Wu G, Wu Y, Lin Y (2023). Pharmaco-proteogenomic characterization of liver cancer organoids for precision oncology. Sci Transl Med.

[64] Zheng C, Zhang B, Li Y, Liu K, Wei W, Liang S (2023). Donafenib and GSK-J4 Synergistically Induce Ferroptosis in Liver Cancer by Upregulating HMOX1 Expression. Adv Sci.

[65] Nuciforo S, Fofana I, Matter MS, Blumer T, Calabrese D, Boldanova T (2018). Organoid Models of Human Liver Cancers Derived from Tumor Needle Biopsies. Cell Rep.

[66] Saito Y, Muramatsu T, Kanai Y, Ojima H, Sukeda A, Hiraoka N (2019). Establishment of Patient-Derived Organoids and Drug Screening for Biliary Tract Carcinoma. Cell Rep.

[67] Koikawa K, Ohuchida K, Ando Y, Kibe S, Nakayama H, Takesue S (2018). Basement membrane destruction by pancreatic stellate cells leads to local invasion in pancreatic ductal adenocarcinoma. Cancer Lett.

[68] Choi D, Gonzalez-Suarez AM, Dumbrava MG, Medlyn M, de Hoyos-Vega JM, Cichocki F (2023). Microfluidic Organoid Cultures Derived from Pancreatic Cancer Biopsies for Personalized Testing of Chemotherapy and Immunotherapy. Adv Sci.

[69] Tsai S, McOlash L, Palen K, Johnson B, Duris C, Yang Q (2018). Development of primary human pancreatic cancer organoids, matched stromal and immune cells and 3D tumor microenvironment models. BMC Cancer.

[70] Grossman JE, Muthuswamy L, Huang L, Akshinthala D, Perea S, Gonzalez RS (2022). Organoid Sensitivity Correlates with Therapeutic Response in Patients with Pancreatic Cancer. Clin Cancer Res.

[71] Huang L, Holtzinger A, Jagan I, BeGora M, Lohse I, Ngai N (2015). Ductal pancreatic cancer modeling and drug screening using human pluripotent stem cell- and patient-derived tumor organoids. Nat Med.

[72] Li Z, Xu H, Yu L, Wang J, Meng Q, Mei H (2022). Patient-derived renal cell carcinoma organoids for personalized cancer therapy. Clin Transl Med.

[73] Fendler A, Bauer D, Busch J, Jung K, Wulf-Goldenberg A, Kunz S (2020). Inhibiting WNT and NOTCH in renal cancer stem cells and the implications for human patients. Nat Commun.

[74] Grassi L, Alfonsi R, Francescangeli F, Signore M, De Angelis ML, Addario A (2019). Organoids as a new model for improving regenerative medicine and cancer personalized therapy in renal diseases. Cell Death Dis.

[75] Maenhoudt N, Defraye C, Boretto M, Jan Z, Heremans R, Boeckx B (2020). Developing Organoids from Ovarian Cancer as Experimental and Preclinical Models. Stem Cell Reports.

[76] Qian J, LeSavage BL, Hubka KM, Ma C, Natarajan S, Eggold JT (2021). Cancer-associated mesothelial cells promote ovarian cancer chemoresistance through paracrine osteopontin signaling. J Clin Invest.

[77] Senkowski W, Gall-Mas L, Falco MM, Li Y, Lavikka K, Kriegbaum MC (2023). A platform for efficient establishment and drug-response profiling of high-grade serous ovarian cancer organoids. Developmental Cell.

[78] Kopper O, de Witte CJ, Lõhmussaar K, Valle-Inclan JE, Hami N, Kester L (2019). An organoid platform for ovarian cancer captures intra- and interpatient heterogeneity. Nat Med.

[79] Hill SJ, Decker B, Roberts EA, Horowitz NS, Muto MG, Worley MJ (2018). Prediction of DNA Repair Inhibitor Response in Short-Term Patient-Derived Ovarian Cancer Organoids. Cancer Discov.

[80] Jacob F, Salinas RD, Zhang DY, Nguyen PTT, Schnoll JG, Wong SZH (2020). A Patient-Derived Glioblastoma Organoid Model and Biobank Recapitulates Inter- and Intra-tumoral Heterogeneity. Cell.

[81] Jacob F, Ming G-l, Song H (2020). Generation and biobanking of patient-derived glioblastoma organoids and their application in CAR T cell testing. Nat Protoc.

[82] Gao D, Vela I, Sboner A, Iaquinta Phillip J, Karthaus Wouter R, Gopalan A (2014). Organoid Cultures Derived from Patients with Advanced Prostate Cancer. Cell.

[83] Boj Sylvia F, Hwang C-I, Baker Lindsey A, Chio Iok In C, Engle Dannielle D, Corbo V (2015). Organoid Models of Human and Mouse Ductal Pancreatic Cancer. Cell.

[84] Gao M, Lin M, Rao M, Thompson H, Hirai K, Choi M (2018). Development of Patient-Derived Gastric Cancer Organoids from Endoscopic Biopsies and Surgical Tissues. Ann Surg Oncol.

[85] Mullen M, Khabele D, Fuh K, Graham E, Fashemi B, van Biljon L Generation and Culturing of High-Grade Serous Ovarian Cancer Patient-Derived Organoids. J Vis Exp. 2023: 10.3791/64878.

[86] Li Y, Gao X, Ni C, Zhao B, Cheng X (2023). The application of patient-derived organoid in the research of lung cancer. Cell Oncol.

[87] DeRose YS, Gligorich KM, Wang G, Georgelas A, Bowman P, Courdy SJ (2013). Patient-derived models of human breast cancer: protocols for in vitro and in vivo applications in tumor biology and translational medicine. Curr Protoc Pharmacol.

[88] Hubert CG, Rivera M, Spangler LC, Wu Q, Mack SC, Prager BC (2016). A Three-Dimensional Organoid Culture System Derived from Human Glioblastomas Recapitulates the Hypoxic Gradients and Cancer Stem Cell Heterogeneity of Tumors Found In Vivo. Cancer Res.

[89] Karakasheva TA, Kijima T, Shimonosono M, Maekawa H, Sahu V, Gabre JT (2020). Generation and Characterization of Patient-Derived Head and Neck, Oral, and Esophageal Cancer Organoids. Curr Protoc Stem Cell Biol.

[90] Hofer M, Lutolf MP (2021). Engineering organoids. Nat Rev Mater.

[91] Broguiere N, Isenmann L, Hirt C, Ringel T, Placzek S, Cavalli E (2018). Growth of Epithelial Organoids in a Defined Hydrogel. Adv Mater.

[92] Sun J, Jabaji Z, Brinkley GJ, Khalil HA, Sears CM, Lei NY (2014). Type I Collagen as an Extracellular Matrix for the In Vitro Growth of Human Small Intestinal Epithelium. PLoS One.

[93] Lindborg BA, Brekke JH, Vegoe AL, Ulrich CB, Haider KT, Subramaniam S (2016). Rapid Induction of Cerebral Organoids From Human Induced Pluripotent Stem Cells Using a Chemically Defined Hydrogel and Defined Cell Culture Medium. Stem Cells Transl Med.

[94] Cruz-Acuña R, Quirós M, Farkas AE, Dedhia PH, Huang S, Siuda D (2017). Synthetic hydrogels for human intestinal organoid generation and colonic wound repair. Nat Cell Biol.

[95] Aisenbrey EA, Murphy WL (2020). Synthetic alternatives to Matrigel. Nat Rev Mater.

[96] Mosquera MJ, Kim S, Bareja R, Fang Z, Cai S, Pan H (2022). Extracellular Matrix in Synthetic Hydrogel-Based Prostate Cancer Organoids Regulate Therapeutic Response to EZH2 and DRD2 Inhibitors. Adv Mater.

[97] Below CR, Kelly J, Brown A, Humphries JD, Hutton C, Xu J (2022). A microenvironment-inspired synthetic three-dimensional model for pancreatic ductal adenocarcinoma organoids. Nat Mater.

[98] Cruz-Acuña R, Kariuki SW, Sugiura K, Karaiskos S, Plaster EM, Loebel C (2023). Engineered hydrogel reveals contribution of matrix mechanics to esophageal adenocarcinoma and identifies matrix-activated therapeutic targets. J Clin Invest.

[99] Toshimitsu K, Takano A, Fujii M, Togasaki K, Matano M, Takahashi S (2022). Organoid screening reveals epigenetic vulnerabilities in human colorectal cancer. Nat Chem Biol.

[100] van de Wetering M, Francies HE, Francis JM, Bounova G, Iorio F, Pronk A (2015). Prospective derivation of a living organoid biobank of colorectal cancer patients. Cell.

[101] Sato T, Vries RG, Snippert HJ, van de Wetering M, Barker N, Stange DE (2009). Single Lgr5 stem cells build crypt-villus structures in vitro without a mesenchymal niche. Nature.

[102] Mou X, Zhang A, He T, Chen R, Zhou F, Yeung TC (2023). Organoid models for Chinese herbal medicine studies. Acta Mater Med.

[103] Sun C-P, Lan H-R, Fang X-L, Yang X-Y, Jin K-T (2022). Organoid Models for Precision Cancer Immunotherapy. Front Immunol.

[104] Dijkstra KK, Cattaneo CM, Weeber F, Chalabi M, van de Haar J, Fanchi LF (2018). Generation of Tumor-Reactive T Cells by Co-culture of Peripheral Blood Lymphocytes and Tumor Organoids. Cell.

[105] Liu J, Li P, Wang L, Li M, Ge Z, Noordam L (2021). Cancer-Associated Fibroblasts Provide a Stromal Niche for Liver Cancer Organoids That Confers Trophic Effects and Therapy Resistance. Cell Mol Gastroenterol Hepatol.

[106] Zhao H, Jiang E, Shang Z (2021). 3D Co-culture of Cancer-Associated Fibroblast with Oral Cancer Organoids. J Dent Res.

[107] Koh V, Chakrabarti J, Torvund M, Steele N, Hawkins JA, Ito Y (2021). Hedgehog transcriptional effector GLI mediates mTOR-Induced PD-L1 expression in gastric cancer organoids. Cancer Lett.

[108] Neal JT, Li X, Zhu J, Giangarra V, Grzeskowiak CL, Ju J (2018). Organoid Modeling of the Tumor Immune Microenvironment. Cell.

[109] Ding S, Hsu C, Wang Z, Natesh NR, Millen R, Negrete M (2022). Patient-derived micro-organospheres enable clinical precision oncology. Cell Stem Cell.

[110] Kheiri S, Yakavets I, Cruickshank J, Ahmadi F, Berman HK, Cescon DW (2024). Microfluidic Platform for Generating and Releasing Patient-Derived Cancer Organoids with Diverse Shapes: Insight into Shape-Dependent Tumor Growth. Adv Mater.

[111] Choi D, Gonzalez-Suarez AM, Dumbrava MG, Medlyn M, de Hoyos-Vega JM, Cichocki F (2024). Microfluidic Organoid Cultures Derived from Pancreatic Cancer Biopsies for Personalized Testing of Chemotherapy and Immunotherapy. Adv Sci (Weinh).

[112] Choi Y-m, Lee H, Ann M, Song M, Rheey J, Jang J (2023). 3D bioprinted vascularized lung cancer organoid models with underlying disease capable of more precise drug evaluation. Biofabrication.

[113] Han J, Jeong HJ, Choi J, Kim H, Kwon T, Myung K (2025). Bioprinted Patient-Derived Organoid Arrays Capture Intrinsic and Extrinsic Tumor Features for Advanced Personalized Medicine. Adv Sci (Weinh).

[114] Huang D-n, Wu F-f, Zhang A-h, Sun H, Wang X-j (2021). Efficacy of berberine in treatment of rheumatoid arthritis: From multiple targets to therapeutic potential. Pharmacol Res.

[115] Hsu CY, Pallathadka H, Gupta J, Ma H, Al-Shukri HHK, Kareem AK (2024). Berberine and berberine nanoformulations in cancer therapy: Focusing on lung cancer. Phytother Res.

[116] Li YF, Gao Y, Liang BW, Cao XQ, Sun ZJ, Yu JH (2020). Patient-derived organoids of non-small cells lung cancer and their application for drug screening. Neoplasma.

[117] Jiang X, Jiang Z, Jiang M, Sun Y (2022). Berberine as a Potential Agent for the Treatment of Colorectal Cancer. Frontiers in Medicine.

[118] Okuno K, Garg R, Yuan Y-C, Tokunaga M, Kinugasa Y, Goel A (2022). Berberine and Oligomeric Proanthocyanidins Exhibit Synergistic Efficacy Through Regulation of PI3K-Akt Signaling Pathway in Colorectal Cancer. Front Oncol.

[119] Devarajan N, Jayaraman S, Mahendra J, Venkatratnam P, Rajagopal P, Palaniappan H (2021). Berberine-A potent chemosensitizer and chemoprotector to conventional cancer therapies. Phytother Res.

[120] Okuno K, Xu C, Pascual-Sabater S, Tokunaga M, Han H, Fillat C (2022). Berberine Overcomes Gemcitabine-Associated Chemoresistance through Regulation of Rap1/PI3K-Akt Signaling in Pancreatic Ductal Adenocarcinoma. Pharmaceuticals.

[121] Arumugam MK, Paal MC, Donohue TM, Ganesan M, Osna NA, Kharbanda KK (2021). Beneficial Effects of Betaine: A Comprehensive Review. Biology.

[122] Kantarjian HM, Talpaz M, Santini V, Murgo A, Cheson B, O'Brien SM (2001). Homoharringtonine: history, current research, and future direction. Cancer.

[123] Li L, Halpert G, Lerner MG, Hu H, Dimitrion P, Weiss MJ (2021). Protein synthesis inhibitor omacetaxine is effective against hepatocellular carcinoma. JCI Insight.

[124] Liang D, Liu L, Zheng Q, Zhao M, Zhang G, Tang S (2023). Chelerythrine chloride inhibits the progression of colorectal cancer by targeting cancer-associated fibroblasts through intervention with WNT10B/β-catenin and TGFβ2/Smad2/3 axis. Phytother Res.

[125] Heng WS, Cheah S-C (2020). Chelerythrine Chloride Downregulates β-Catenin and Inhibits Stem Cell Properties of Non-Small Cell Lung Carcinoma. Molecules.

[126] Hu Y, Yu X, Yang L, Xue G, Wei Q, Han Z (2024). Research progress on the antitumor effects of harmine. Front Oncol.

[127] Zhu J, Zhu H, Zhu Q, Xu SL, Xiao L, Zhang MY (2024). The roles of autophagy, ferroptosis and pyroptosis in the anti-ovarian cancer mechanism of harmine and their crosstalk. Sci Rep.

[128] Cui Z, Crane J, Xie H, Jin X, Zhen G, Li C (2016). Halofuginone attenuates osteoarthritis by inhibition of TGF-β activity and H-type vessel formation in subchondral bone. Ann Rheum Dis.

[129] Gill J, Sharma A (2022). Prospects of halofuginone as an antiprotozoal drug scaffold. Drug Discovery Today.

[130] Li H, Zhang Y, Lan X, Yu J, Yang C, Sun Z (2021). Halofuginone Sensitizes Lung Cancer Organoids to Cisplatin via Suppressing PI3K/AKT and MAPK Signaling Pathways. Front Cell Dev Biol.

[131] Fu R, Wang X, Hu Y, Du H, Dong B, Ao S (2019). Solamargine inhibits gastric cancer progression by regulating the expression of lncNEAT1_2 via the MAPK signaling pathway. Int J Oncol.

[132] Yin S, Jin W, Qiu Y, Fu L, Wang T, Yu H (2022). Solamargine induces hepatocellular carcinoma cell apoptosis and autophagy via inhibiting LIF/miR-192-5p/CYR61/Akt signaling pathways and eliciting immunostimulatory tumor microenvironment. J Hematol Oncol.

[133] Han Y, Shi J, Xu Z, Zhang Y, Cao X, Yu J (2022). Identification of solamargine as a cisplatin sensitizer through phenotypical screening in cisplatin-resistant NSCLC organoids. Front Pharmacol.

[134] Zhou L, Hong G, Li S, Liu Q, Song F, Zhao J (2020). Fangchinoline protects against bone loss in OVX mice via inhibiting osteoclast formation, bone resorption and RANKL-induced signaling. Int J Biol Sci.

[135] Harrison PT, Vyse S, Huang PH (2020). Rare epidermal growth factor receptor (EGFR) mutations in non-small cell lung cancer. Semin Cancer Biol.

[136] Chen B, Song Y, Zhan Y, Zhou S, Ke J, Ao W (2022). Fangchinoline inhibits non-small cell lung cancer metastasis by reversing epithelial-mesenchymal transition and suppressing the cytosolic ROS-related Akt-mTOR signaling pathway. Cancer Lett.

[137] Min YD, Kwon HC, Yang MC, Lee KH, Choi SU, Lee KR (2007). Isolation of limonoids and alkaloids from Phellodendron amurense and their multidrug resistance (MDR) reversal activity. Arch Pharm Res.

[138] Wang Y, Li Y-J, Huang X-H, Zheng C-C, Yin X-F, Li B (2018). Liensinine perchlorate inhibits colorectal cancer tumorigenesis by inducing mitochondrial dysfunction and apoptosis. Food & Function.

[139] He YQ, Ma ZY, Wei XM, Liu DJ, Du BZ, Yao BH (2011). Honatisine, a novel diterpenoid alkaloid, and six known alkaloids from Delphinium honanense and their cytotoxic activity. Chem Biodivers.

[140] Li Z, Sai K, Ma G, Chen F, Xu X, Chen L (2024). Diterpenoid honatisine overcomes temozolomide resistance in glioblastoma by inducing mitonuclear protein imbalance through disruption of TFAM-mediated mtDNA transcription. Phytomedicine.

[141] Zeng Z-w, Chen D, Chen L, He B, Li Y (2023). A comprehensive overview of Artemisinin and its derivatives as anticancer agents. Eur J Med Chem.

[142] Dai X, Zhang X, Chen W, Chen Y, Zhang Q, Mo S (2021). Dihydroartemisinin: A Potential Natural Anticancer Drug. Int J Biol Sci.

[143] Yang Z, Zhou Z, Meng Q, Chen Z, Yun L, Jiang J (2024). Dihydroartemisinin Sensitizes Lung Cancer Cells to Cisplatin Treatment by Upregulating ZIP14 Expression and Inducing Ferroptosis. Cancer Med.

[144] Dai Y, Chen S-R, Chai L, Zhao J, Wang Y, Wang Y (2018). Overview of pharmacological activities of Andrographis paniculata and its major compound andrographolide. Crit Rev Food Sci Nutr.

[145] Sharma P, Shimura T, Banwait JK, Goel A (2020). Andrographis-mediated chemosensitization through activation of ferroptosis and suppression of β-catenin/Wnt-signaling pathways in colorectal cancer. Carcinogenesis.

[146] Shimura T, Sharma P, Sharma GG, Banwait JK, Goel A (2021). Enhanced anti-cancer activity of andrographis with oligomeric proanthocyanidins through activation of metabolic and ferroptosis pathways in colorectal cancer. Sci Rep.

[147] Song M, Wang X, Luo Y, Liu Z, Tan W, Ye P (2020). Cantharidin suppresses gastric cancer cell migration/invasion by inhibiting the PI3K/Akt signaling pathway via CCAT1. Chemico-Biological Interactions.

[148] Yang T, Yu R, Cheng C, Huo J, Gong Z, Cao H (2023). Cantharidin induces apoptosis of human triple negative breast cancer cells through mir-607-mediated downregulation of EGFR. J Transl Med.

[149] He Z, Hu Y, Niu Z, Zhong K, Liu T, Yang M (2023). A review of pharmacokinetic and pharmacological properties of asiaticoside, a major active constituent of Centella asiatica (L.) Urb. J Ethnopharmacol.

[150] Guo Y, Xu J, Jia Y, Tian Y, Zhang Y, Zhang J (2024). Asiaticoside modulates human NK cell functional fate by mediating metabolic flexibility in the tumor microenvironment. Phytomedicine.

[151] Park D, Jung JH, Ko HM, Jee W, Kim H, Jang H-J (2022). Antitumor Effect of Cycloastragenol in Colon Cancer Cells via p53 Activation. Int J Mol Sci.

[152] Deng G, Zhou L, Wang B, Sun X, Zhang Q, Chen H (2022). Targeting cathepsin B by cycloastragenol enhances antitumor immunity of CD8 T cells via inhibiting MHC-I degradation. J Immunother Cancer.

[153] Acton N, Klayman DL (1985). Artemisitene, a New Sesquiterpene Lactone Endoperoxide from Artemisia annua. Planta Med.

[154] Dong T, Li C, Wang X, Dian L, Zhang X, Li L (2015). Ainsliadimer A selectively inhibits IKKα/β by covalently binding a conserved cysteine. Nat Commun.

[155] Lv C, Huang Y, Wang Q, Wang C, Hu H, Zhang H (2023). Ainsliadimer A induces ROS-mediated apoptosis in colorectal cancer cells via directly targeting peroxiredoxin 1 and 2. Cell Chem Biol.

[156] Modzelewska A, Sur S, Kumar SK, Khan SR (2005). Sesquiterpenes: natural products that decrease cancer growth. Curr Med Chem Anticancer Agents.

[157] Cui P, Chen F, Ma G, Liu W, Chen L, Wang S (2022). Oxyphyllanene B overcomes temozolomide resistance in glioblastoma: Structure-activity relationship and mitochondria-associated ER membrane dysfunction. Phytomedicine.

[158] Carletti I, Long C, Funel C, Amade P (2003). Yardenone A and B: new cytotoxic triterpenes from the Indian Ocean sponge Axinella cf. bidderi. J Nat Prod.

[159] Dai J, Fishback JA, Zhou Y-D, Nagle DG (2006). Sodwanone and Yardenone Triterpenes from a South African Species of the Marine Sponge Axinella Inhibit Hypoxia-Inducible Factor-1 (HIF-1) Activation in Both Breast and Prostate Tumor Cells. J Nat Prod.

[160] Peng S, Guo Y, Irondelle M, Mazzu A, Kahi M, Ferreira Montenegro P (2024). The marine-derived HIF-1α inhibitor, Yardenone 2, reduces prostate cancer cell proliferation by targeting HIF-1 target genes. Cell Mol Biol Lett.

[161] Tomeh MA, Hadianamrei R, Zhao X (2019). A Review of Curcumin and Its Derivatives as Anticancer Agents. Int J Mol Sci.

[162] Miyazaki K, Xu C, Shimada M, Goel A (2023). Curcumin and Andrographis Exhibit Anti-Tumor Effects in Colorectal Cancer via Activation of Ferroptosis and Dual Suppression of Glutathione Peroxidase-4 and Ferroptosis Suppressor Protein-1. Pharmaceuticals.

[163] Ye HS, Gao HF, Li H, Nie JH, Li TT, Lu MD (2022). Higher efficacy of resveratrol against advanced breast cancer organoids: A comparison with that of clinically relevant drugs. Phytother Res.

[164] Mukund V, Mukund D, Sharma V, Mannarapu M, Alam A (2017). Genistein: Its role in metabolic diseases and cancer. Crit Rev Oncol Hematol.

[165] Cheng J, Xie W, Chen Y, Sun Y, Gong L, Wang H (2024). Drug resistance mechanisms in dopamine agonist-resistant prolactin pituitary neuroendocrine tumors and exploration for new drugs. Drug Resist Updat.

[166] Imran M, Rauf A, Abu-Izneid T, Nadeem M, Shariati MA, Khan IA (2019). Luteolin, a flavonoid, as an anticancer agent: A review. Biomed Pharmacother.

[167] Hao X, Zu M, Ning J, Zhou X, Gong Y, Han X (2023). Antitumor effect of luteolin proven by patient-derived organoids of gastric cancer. Phytother Res.

[168] Yi C, Li G, Ivanov DN, Wang Z, Velasco MX, Hernández G (2018). Luteolin inhibits Musashi1 binding to RNA and disrupts cancer phenotypes in glioblastoma cells. RNA Biology.

[169] Fatima R, Soni P, Sharma M, Prasher P, Kaverikana R, Mangalpady SS (2025). Fisetin as a chemoprotective and chemotherapeutic agent: mechanistic insights and future directions in cancer therapy. Med Oncol.

[170] Kim N, Kwon J, Shin US, Jung J (2023). Fisetin induces the upregulation of AKAP12 mRNA and anti-angiogenesis in a patient-derived organoid xenograft model. Biomed Pharmacother.

[171] Luo P, An Y, He J, Xing X, Zhang Q, Liu X (2024). Icaritin with autophagy/mitophagy inhibitors synergistically enhances anticancer efficacy and apoptotic effects through PINK1/Parkin-mediated mitophagy in hepatocellular carcinoma. Cancer Lett.

[172] Kang FP, Chen ZW, Liao CY, Wu YD, Li G, Xie CK (2024). Escherichia coli-Induced cGLIS3-Mediated Stress Granules Activate the NF-κB Pathway to Promote Intrahepatic Cholangiocarcinoma Progression. Adv Sci (Weinh).

[173] Ravindranathan P, Pasham D, Goel A (2019). Oligomeric proanthocyanidins (OPCs) from grape seed extract suppress the activity of ABC transporters in overcoming chemoresistance in colorectal cancer cells. Carcinogenesis.

[174] Toden S, Ravindranathan P, Gu J, Cardenas J, Yuchang M, Goel A (2018). Oligomeric proanthocyanidins (OPCs) target cancer stem-like cells and suppress tumor organoid formation in colorectal cancer. Sci Rep.

[175] Ravindranathan P, Pasham D, Balaji U, Cardenas J, Gu J, Toden S (2018). Mechanistic insights into anticancer properties of oligomeric proanthocyanidins from grape seeds in colorectal cancer. Carcinogenesis.

[176] Buonfiglio D, Hummer DL, Armstrong A, Christopher Ehlen J, DeBruyne JP (2020). Angelman syndrome and melatonin: What can they teach us about sleep regulation. J Pineal Res.

[177] Wang L, Wang C, Choi WS (2022). Use of Melatonin in Cancer Treatment: Where Are We?. Int J Mol Sci.

[178] Zhao Y, Wang C, Goel A (2022). A combined treatment with melatonin and andrographis promotes autophagy and anticancer activity in colorectal cancer. Carcinogenesis.

[179] Sheikhnia F, Rashidi V, Maghsoudi H, Majidinia M (2023). Potential anticancer properties and mechanisms of thymoquinone in colorectal cancer. Cancer Cell Int.

[180] Al Bitar S, Ballout F, Monzer A, Kanso M, Saheb N, Mukherji D (2022). Thymoquinone Radiosensitizes Human Colorectal Cancer Cells in 2D and 3D Culture Models. Cancers.

[181] Chu Y, Yuan Q, Jiang H, Wu L, Xie Y, Zhang X (2024). A comprehensive review of the anticancer effects of decursin. Front Pharmacol.

[182] Kim S, Lee SI, Kim N, Joo M, Lee KH, Lee MW (2021). Decursin inhibits cell growth and autophagic flux in gastric cancer via suppression of cathepsin C. Am J Cancer Res.

[183] Merarchi M, Jung YY, Fan L, Sethi G, Ahn KS (2019). A Brief Overview of the Antitumoral Actions of Leelamine. Biomedicines.

[184] Ma J, Zhao J, Wu Z, Tan J, Xu M, Ye W (2024). Dehydroabietylamine exerts antitumor effects by affecting nucleotide metabolism in gastric cancer. Carcinogenesis.

[185] Zhao L, Zhang Y, Li Y, Li C, Shi K, Zhang K (2022). Therapeutic effects of ginseng and ginsenosides on colorectal cancer. Food Funct.

[186] Okuno K, Pratama MY, Li J, Tokunaga M, Wang X, Kinugasa Y (2023). Ginseng mediates its anticancer activity by inhibiting the expression of DNMTs and reactivating methylation-silenced genes in colorectal cancer. Carcinogenesis.

[187] Jiménez MC, Prieto K, Lasso P, Gutiérrez M, Rodriguez-Pardo V, Fiorentino S (2023). Plant extract from Caesalpinia spinosa inhibits cancer-associated fibroblast-like cells generation and function in a tumor microenvironment model. Heliyon.

[188] Urueña C, Sandoval TA, Lasso P, Tawil M, Barreto A, Torregrosa L (2020). Evaluation of chemotherapy and P2Et extract combination in ex-vivo derived tumor mammospheres from breast cancer patients. Sci Rep.

[189] Lo YH, Kolahi KS, Du Y, Chang CY, Krokhotin A, Nair A (2021). A CRISPR/Cas9-Engineered ARID1A-Deficient Human Gastric Cancer Organoid Model Reveals Essential and Nonessential Modes of Oncogenic Transformation. Cancer Discov.

[190] Tuveson D, Clevers H (2019). Cancer modeling meets human organoid technology. Science.

[191] Yan HHN, Siu HC, Law S, Ho SL, Yue SSK, Tsui WY (2018). A Comprehensive Human Gastric Cancer Organoid Biobank Captures Tumor Subtype Heterogeneity and Enables Therapeutic Screening. Cell Stem Cell.

[192] Calandrini C, Schutgens F, Oka R, Margaritis T, Candelli T, Mathijsen L (2020). An organoid biobank for childhood kidney cancers that captures disease and tissue heterogeneity. Nat Commun.

[193] Kong J, Lee H, Kim D, Han SK, Ha D, Shin K (2020). Network-based machine learning in colorectal and bladder organoid models predicts anti-cancer drug efficacy in patients. Nat Commun.

[194] Wang H, Li X, You X, Zhao G (2024). Harnessing the power of artificial intelligence for human living organoid research. Bioact Mater.

